# Are more charging piles imperative to future electrified transportation system?

**DOI:** 10.1016/j.fmre.2022.12.006

**Published:** 2022-12-24

**Authors:** Xiaobo Qu, Hongzhang Shao, Shuaian Wang, Yunpeng Wang

**Affiliations:** a School of Vehicle and Mobility, Tsinghua University, Beijing 100084, China; b H. Milton Stewart School of Industrial and Systems Engineering, Georgia Institute of Technology, Atlanta 30067, USA; c Faculty of Business, The Hong Kong Polytechnic University, Hong Kong SAR, China; d School of Transportation Science and Engineering, Beihang University, Beijing 100191, China

**Keywords:** Vehicle-to-vehicle charging, Transportation electrification, Charging pile network, Electric vehicles, Vehicle modularization

## Abstract

Scholars and practitioners believe that the large-scale deployment of charging piles is imperative to our future electric transportation systems. Major economies ambitiously install charging pile networks, with massive construction spending, maintenance costs, and urban space occupation. However, recent developments in technology may significantly reduce the necessary charging capacity required by the system. This paper develops a linear programming model to characterize the effects of likely scenarios where vehicle-to-vehicle (V2V) charging is available via vehicle modularization or wireless charging. Specifically, we consider scenarios in which vehicles can transmit energy to each other (coordinated by a central platform) while traveling closely on the same road. We first estimate the number of charging piles needed for completing the travel plan of 73 cars from data, assuming a battery capacity of 400 km’s range and no V2V charging. Our results show that once V2V charging technologies with an efficiency of 50% are available, more than 2/3 of the charging piles investment would be wasted. Additionally, if the efficiency of V2V charging increases to 75%, we can easily reduce the battery capacity of vehicles to 200 km, which will reduce production costs and improve energy efficiency. These results may reveal us an alternative pathway towards transportation electrification.

## 1 Introduction

Electric vehicles (EVs) have been considered an essential solution to the greenhouse gas emissions from the transportation sector [1], which accounted for 20.3% of emissions globally [2]. Compared with conventional vehicles, they produce considerably lower emissions over their life span [3]. The adoption of electric vehicles will become even more urgent, considering that we have witnessed a constant increase in emissions from the transportation sector since 2013. In comparison, the carbon footprint generated from the industry and agriculture sectors has substantially decreased over the past years. It has been a consensus that our future transportation systems must be electrified [4]. According to the American Jobs Plan [5], the United States infrastructure bill is investing 174 billion dollars in building an electrified transportation network, with a significant component in constructing charging piles. China had electrified 60% of its buses by October 2020 [6], and had promised that 20% of its motor vehicle market share would be from pure electric vehicles by 2025 [7]. The BloombergNEF (BNEF) consultancy in London estimates that in 2035, at least half of the global passenger-vehicle sales will be electric [4].

Given the limited driving range and long charging time of current electric vehicles, most people believe it would be challenging to adopt more electric vehicles without a lot more charging piles [8], [9]. Practitioners and researchers have projected that Europe will need 65 million charging piles by 2035 [10]. Taking the average estimated cost of $4855 for a Level 2 commercial charger [11], Europe will need to invest over $300 billion. The construction, maintenance, and management of these charging piles can be even more expensive, as they will likely be in urban areas where demands are high, and land is scarce. Researchers also predict that the idle rate of charging piles will be high [9]. At the same time, carmakers are equipping electric vehicles with increasingly larger batteries in response to the range anxiety and the shortage of charging piles. However, larger batteries are more expensive. They are also heavier and further increase energy consumption [12]. For example, Tesla Model S P85 (MF) has a battery capacity of 85 kWh. The battery alone weighs as high as 540 kg, while the vehicle’s total curb weight is only 2200 kg.

**Fig. 1:**
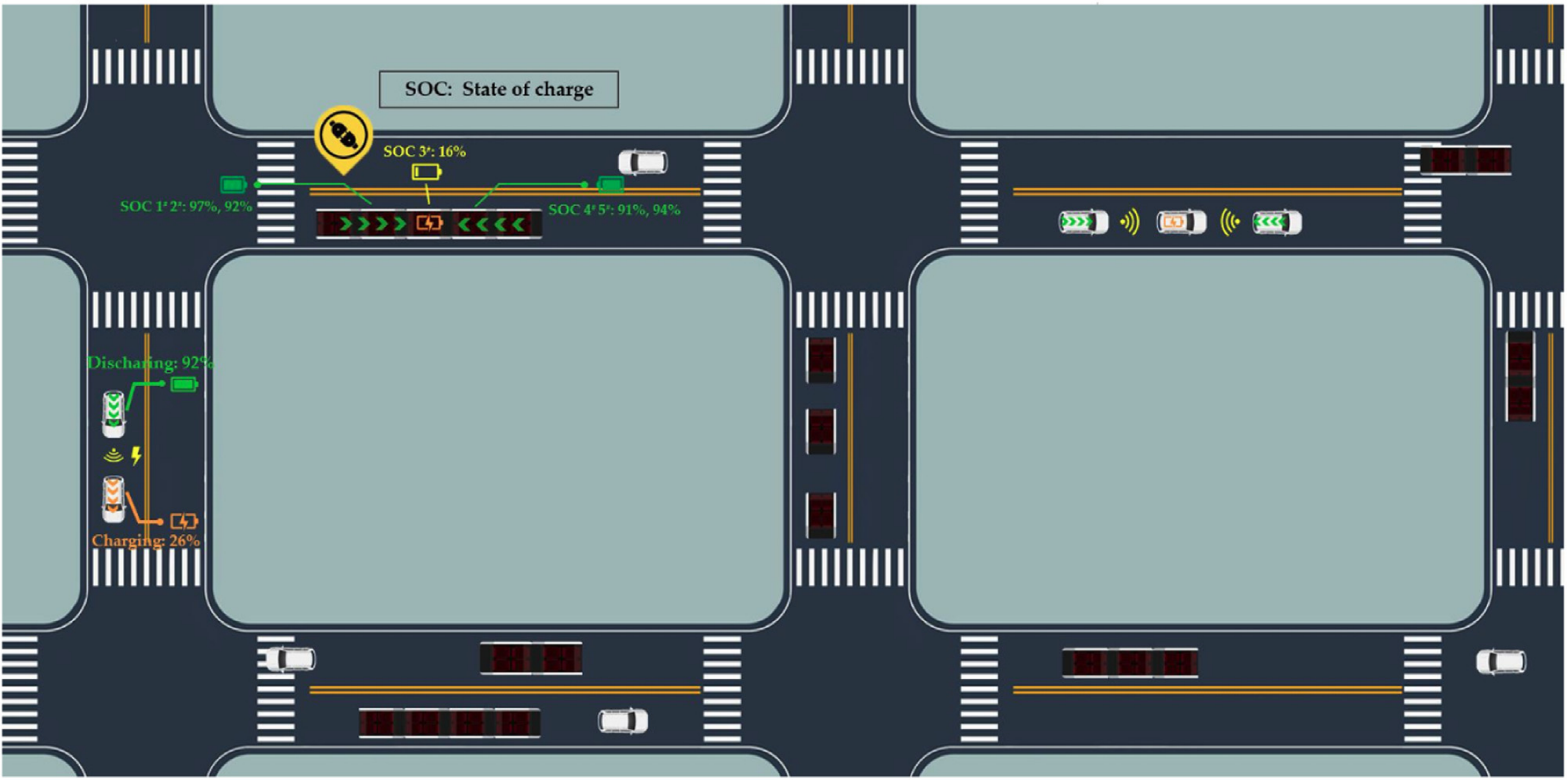
**V2V wireless charging and modular buses in an illustrative network**.

**Fig. 2:**
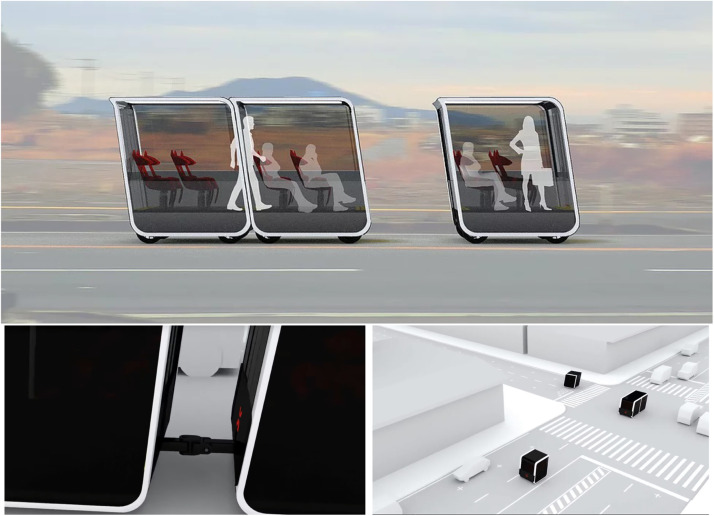
**Modular buses by the next future transportation** (https://www.next-future-mobility.com/).

This paper discusses a different but essential aspect in assessing the infrastructure requirements for electrifying the transportation system. We consider scenarios in which vehicles can transmit energy to other vehicles through a physical connection or wireless charging technology (see Fig. 1). Research has demonstrated feasibility in both vehicle-to-vehicle charging technologies. Wireless charging technologies for electric vehicles have also been in use since 2011. Researchers have estimated a charging efficiency of around 90% with a distance of 1 m (3.28 feet) [13]. While most researchers are focusing on infrastructure-to-vehicle wireless charging, the same technology can be used for wireless charging between vehicles in the same platoon as it matures. On the other hand, the feasibility of V2V charging through a physical connection can be demonstrated through the development of modular vehicle (MV) technologies. As shown in Fig. 2, MV technologies allow vehicles to connect physically and rebalance their batteries in motion. Although large-scale adoption is challenging, engineers are already conducting pilot experiments of MVs in the Middle East and Europe [14]. We refer the readers to Chakraborty et al. [15] for more discussion on the engineering aspect of V2V charging.

We consider a scenario in which a centralized energy cloud system coordinates the transmission of energy between vehicles as they travel close to each other (e.g., on the same road). In this scenario, vehicles will continue to follow their existing travel plans and patterns. The energy cloud system can therefore coexist with other systems that control vehicle travel without interfering with them. However, vehicles will give permission to the centralized system to (1) know their travel plans for planning purposes, (2) arrange them into the same platoon while they are traveling close together, and (3) instruct them to transmit energy to or from other vehicles. We are interested in the possible reduction of costs in infrastructure (e.g. charging piles) and manufacturing (e.g. car batteries) when such a system is introduced. Although few people think of such a scenario, our models and analysis demonstrated that it may fundamentally change our path to transportation electrification. Without considering this possibility, people are likely to overreact to the growing market share of electric vehicles. Right now, vast networks of charging piles and large batteries are almost universally acknowledged to be necessary. However, they may soon become useless burdens.

Previous research on the management of electric vehicles has focused on the relationship between EVs and the electricity smart grid [16], [17], [18], [19], [20] and the effect of EV on the carsharing market [21], [22]. A few studies have examined EV charging infrastructure planning in the context of existing charging technologies [23], [24], [25], [26], whereas we consider a fundamentally different technological pathway. More concretely, we provide a strategical examination of whether investing in an extensive network of charging piles and equipping electric vehicles with large batteries are imperative, with or without V2V charging.

Closet to our paper is the one by Chakraborty et al. [15]. They studied the operational problems, including scheduling, paring, and rerouting of vehicles within a V2V charging system. The major difference between our work and their work is that they did not consider the travel plans that vehicles need to follow. Instead, their control system only completes a set of exogenous charging requests, assuming that all vehicles were willing to give up control in choosing their travel schedule, route, etc. Thus, in their system, vehicles need to reroute themselves, reschedule their travel plans, and take detours, in order to take or give energy. An analogy to this approach is to pass along energy to a vehicle through a “relay race” using a sequence of charging piles with batteries and wheels on them. While the additional waste of time and energy can be partially reduced as they introduce dedicated charging vehicles with giant batteries, putting these vehicles into the system requires additional investments. (The closed-loop effect that rerouting vehicles create additional charging needs is also ignored.) In contrast, our approach is more applicable as it relies on existing infrastructure and only provides a platform for energy sharing among vehicles without alternating their travel plans. Additionally, our work focuses on finding the largest possible reduction in infrastructure and manufacturing costs, while ref. [15] focused on control problems in the system.

The remainder of the paper is organized as follows. In Section 2, we develop models that calculate the minimum installation cost of charging stations and the minimum size of vehicle batteries with and without V2V charging technologies. In Section 3, we report the results of numerical studies using data in Shenzhen, China. In Section 4, we discuss the managerial implications of the results. We conclude the paper in Section 5. For the simplicity of tracking notation, we summarize all of the notation in Table 1.

**Table 1:** **Notations**.

Symbol	Category	Definition
N	data	number of vehicles
T	data	number of time periods
$V_{it}$	data	average velocity of vehicle i∈N during
		time period t∈{1,…,T}
$I_{lit}$	data	indicator if vehicle i∈N can charge from
		station l∈L during time t∈{1,…,T}
$J_{lit}$	data	indicator if vehicle i∈N can charge
		vehicle j∈N during time t∈{1,…,T}
N	data	set of all vehicles
L	data	set of (potential) locations of charging stations
τ	parameter	length of time periods
$\bar{\beta }$	parameter	battery size (maximum battery level)
$\underset{‾}{\beta }$	parameter	safety (minimum) level of battery
μ	parameter	maximum charging rate of vehicles
$\bar{\nu }$	parameter	maximum (giving) V2V charging rate
ν	parameter	maximum (receiving) V2V charging rate
θ	parameter	V2V transferring efficiency of electricity
γ	parameter	(maximum) total charging capacity of all stations
$b_{0t}$	variable	initial battery level of vehicle i∈N
$b_{it}$	variable	battery level of vehicle i∈N at the end of
		time period t∈{1,…,T}
$u_{it}$	variable	battery level refilled to vehicle i∈N during
		time period t∈{1,…,T}
$c_{l}$	variable	charging capacity of station l∈L
$v_{ijt}$	variable	vehicle i∈N consumes to recharge vehicle j∈N
		time period t∈{1,…,T}
z	variable	battery size (when $\underset{‾}{\beta }$ becomes a decision variable)

## 2 Model

Consider a region with N gasoline vehicles, and let N denote the set of all vehicles. Latitude, longitude, and speed data are collected for these vehicles over T time periods with an equal length τ (e.g., 2 min). Suppose that the vehicles are to be replaced by electric vehicles. Using the data, we aim to estimate the minimal costs and requirements, such as the installation cost of new charging stations and the size of vehicle batteries, for this replacement to be possible. We also aim to determine whether V2V charging can reduce costs and requirements.

We formulate optimization models to find the minimal costs and requirements to ensure that every electric vehicle can complete its travel plan as specified by the dataset. Specifically, vehicles consume electricity when moving but can refill their batteries at a charging station. The optimization models allocate resources to ensure that the battery levels of all vehicles stay above a pre-determined safety level $\underset{‾}{\beta }$ throughout the planning horizon. (As a convention, we use lowercase English letters for decision variables and indices, uppercase English letters for input data, and Greek letters for constant parameters.) We assume that all vehicles have the same physical properties (i.e., they are identical in every aspect, including battery capacity, battery consumption rate, and maximum charging rate). Let $V_{it}$ denote the average velocity of vehicle i during time period t for each i∈N and t∈{1,…,T}. Let $b_{it}$ denote the battery level of vehicle i at the end of time period t, and let $b_{i0}$ denote the initial battery level of vehicle i (for convenience, we use the index t∈{1,…,T} to refer to both the time period and the end of that time period). Here, we measure battery level by the distance a vehicle can travel without having to stop to charge. Thus, each vehicle i consumes $\tau V_{it}$ battery power during time period t. In other words, the energy consumption of a vehicle is proportional to its travel distance (or, equivalently, its average speed during a time period). If a vehicle is not traveling, it does not consume battery power.

Remark 1 The task network is at the very center of our model formulation. In this paper, we assume that all tasks need to be completed. This assumption may seem rigid and can be relaxed in several ways. For example, we can allow vehicles to take detours as long as they can get to their destination in time. We can also ask a certain percentage of vehicles to give up their travel plans and spend time serving other vehicles.

One reason that we keep this rigid assumption, is that we do not want to make the results too optimistic. Recall we assumed that vehicles are coordinated by a centralized system. They need to give their travel plans to the system in advance and follow its order in platooning and transmitting energy with other vehicles. If vehicles need to sacrifice their time by taking detours and making themselves into a moving power bank, it is much less likely that the system can attract enough users and become useful.

Another benefit this assumption gives is simplicity. While introducing relaxations such as detours solves this one concern, it creates many other concerns. The percentage of tasks that need to be completed, as well as the number of detours that vehicles can take, are all new parameters that need to be chosen. Without good data and reasoning, they would have to be chosen subjectively. Thus, making a model more flexible does not necessarily make its results more reliable. (There are special cases, however, that this assumption needs to be relaxed. A brief discussion is provided in Section 4.)

### 2.1 Minimal installation cost of charging stations

We measure the installation cost by the total charging capacity of all charging stations instead of the integral number of charging piles for computational tractability. We assume that there are infinite positions at each charging station, such that all vehicles in the station can charge from it, as long as the total charging rate does not exceed its capacity. Thus, we characterize the charging capacity as the total amount of battery power vehicles can refill from the stations within each time period. Let L denote the set of potential locations of charging stations. Let $c_{l}$ denote the charging capacity of station l∈L. For each l∈L and i∈N, let $I_{lit}\in \left\{0,1\right\}$ denote the indicator that takes value 1 if vehicle i can recharge itself at location l during time period t, and 0 otherwise. Here both L and $I:=(I_{lit}:\;l\in L,\;i\in N,\;t\in \left\{1,\ldots ,T\right\})$ are inputs that can be obtained from the data by applying certain rules. For example, L can be the set of all locations at which a vehicle has parked. If external data are available, L can also be the set of all parking lots, gas stations, and residential areas within the region. Note that each vehicle can charge from at most one station during each time period. Thus, we need $\sum_{l\in L}I_{lit}\leq 1$ for every i∈N and t∈{1,…,T}.

Vehicles refill batteries from charging stations. For each time period t∈{1,…,T} and each vehicle i∈N, we let $u_{it}$ denote the battery power recharged to vehicle i during time period t (the location of the charging station is indicated by $I_{lit}$). Let μ denote the maximum charging rate of vehicles (i.e., maximum battery power a vehicle can refill within a time period) from stations. That is, $u_{it}\leq \mu$ for any i∈N and t∈{1,…,T}. In addition, let $\bar{\beta }$ denote the battery size (maximum battery level) of vehicles. That is, $b_{it}\leq \bar{\beta }$ for any i∈N and t∈{0,…,T}.

Vehicles can also transfer electricity among one another. For each time period t∈{1,…,T} and each pair of vehicles i,j∈N, we let $v_{ijt}$ denote the battery level vehicle i consumes to recharge the battery of vehicle j during time t, and $J_{ijt}\in \left\{0,1\right\}$ be the indicator that takes value 1 if such a recharge can happen (e.g. vehicle i and j can be arranged into the same platoon) during time period t, and 0 otherwise. Note that $I_{lit}$ and $J_{ijt}$ capture the spatial locations of vehicles in different time periods. We let θ∈[0,1] denote the transferring efficiency. If vehicle i consumes battery power of $v_{ijt}$ to recharge the battery of vehicle j during time period t, then vehicle j restores the battery level of $\theta v_{ijt}$. Thus, we can *disable* V2V charging by setting θ=0. Additionally, let ν denote the maximum (receiving) charging rate vehicles can receive from other vehicles. That is, for any i∈N and t∈{1,…,T}, we need $\sum_{j\in N}\theta v_{jit}\leq \nu$. Similarly, let $\bar{\nu }$ denote the maximum (giving) charging rate vehicles can provide for other vehicles. That is, for any i∈N and t∈{1,…,T}, we need $\sum_{j\in N}v_{ijt}\leq \bar{\nu }$.

We can find the minimum total charging capacity of all stations by solving the following model:

($P_{1}$) $$\begin{array}{cc} \min_{b,\;c,\;u,\;v}\; & \sum_{l\in L}c_{l} \end{array}$$

(1a) $$\begin{array}{cc} \;\;\text{s.t.}\; & b_{\mathrm{it}}+\tau V_{\mathrm{it}}+\sum_{j\in N}J_{\mathrm{ijt}}v_{\mathrm{ijt}}\\ & \;\;\;=\;b_{\mathrm{it}-1}+\left(\sum_{l\in L}I_{\mathrm{lit}}\right)u_{\mathrm{it}}+\sum_{j\in N}\theta J_{\mathrm{jit}}v_{\mathrm{jit}}\\ & \;\forall \;i\in N\;\;t=1,\cdots ,T \end{array}$$

(1b) $$\begin{array}{cc} & \sum_{i\in N}I_{\mathrm{lit}}u_{\mathrm{it}}\;\;\leq \;\;c_{l}\\ & \;\;\forall \;l\in L\;\;t=1,\cdots ,T \end{array}$$

(1c) $$\begin{array}{cc} & \;\;\sum_{j\in N}\theta v_{jit}\;\;\leq \;\;\nu \\ & \;\;\;\;\forall \;i\in N\;\;t=1,\ldots ,T \end{array}$$

(1d) $$\begin{array}{cc} & \;\;\sum_{j\in N}v_{ijt}\;\;\leq \;\;\bar{\nu }\\ & \;\;\;\forall \;i\in N\;\;t=1,\ldots ,T \end{array}$$

(1e) $$\begin{array}{cc} & \;\;\sum_{i\in N}b_{i0}\;\;\leq \;\;\sum_{i\in N}b_{iT} \end{array}$$

(1f) $$\begin{array}{cc} & \;\;0\;\;\leq \;\;u\;\;\leq \;\;\mu \end{array}$$

(1g) $$\begin{array}{cc} & \;\;\underset{‾}{\beta }\;\;\leq \;\;b\;\;\leq \;\;\bar{\beta } \end{array}$$

(1h) $$\begin{array}{cc} & \;\;v\;\;\geq \;\;0 \end{array}$$

(1i) $$\begin{array}{cc} & \;\;c\;\;\geq \;\;0. \end{array}$$

In problem (P1), constraint (1a) ensures that vehicles follow the rules of consuming electricity with regards to moving and refilling electricity from charging stations (while parked) or other vehicles. Constraint (1b) then checks that the total charging rate to all vehicles from each station does not exceed its capacity (recall that $\sum_{l\in L}I_{lit}\leq 1$). Note that in problem (P1), battery levels, as well as initial battery levels, are decision variables. The idea here is that we assume that drivers are able to determine the best travel and recharge plans for themselves. We then use constraint (1e) to prevent “overfitting” and free ourselves from the impact of the conditions of initial battery levels. Intuitively, drivers should still be capable of future travel at the end of the planning horizon. Thus, the total ending battery level should be greater or equal to the total initial battery level.

A few remarks here: As a network flow problem, the value of variable v forms a tree structure at optimality. Specifically, the transfer of electricity should not contain circles (otherwise, electricity is wasted). Here problem (P1) allows electricity to be transferred between multiple vehicles simultaneously (i.e., a vehicle can receive electricity from multiple givers and can transmit electricity to multiple receivers during the same time period). In cases in which electricity can only be transferred between vehicles one pair at a time, we can perform “time-division multiplexing” by dividing each time period into smaller sub-periods and then work with different charging pairs during different sub-periods. Thus, the solution from problem (P1) will still be valid. Also, when data are available, it is more realistic to assume that there are already charging stations installed in the region. In this case, we can add constraints $c_{l}\geq C_{l}\;\;l\in L$ to problem (P1), where for each l∈L, $C_{l}$ is the capacity that is already installed at location l. We then simply need to subtract $\sum_{l\in L}C_{l}$ from the objective value, because this existing capacity is already installed.

### 2.2 Minimum size of vehicle batteries

The values of parameters μ, ν, $\bar{\nu }$, θ, $\underset{‾}{\beta }$, and $\bar{\beta }$ may have strong impacts on the solution of problem (P1) and problem (P2). Whereas charging rates μ, ν, $\bar{\nu }$ and charging efficiency θ are relatively exogenous in practice, battery size $\bar{\beta }$ can be controlled. However, battery accounts for a large portion of the cost of producing electric vehicles, and reducing the battery size can be profitable for vehicle manufacturers. Thus, it is necessary to establish the minimum size of vehicle batteries, considering the travel plans of vehicles and the capacities of charging stations.

Let z denote the battery size of vehicles. Let γ denote the total charging capacity. Then the minimum size of vehicle batteries can be found by solving the following model:

($P_{2}$) $$\begin{array}{cc} \min_{b,\;c,\;u,\;v,\;z}\; & z \end{array}$$

(2a) $$\begin{array}{cc} \;\;\text{s.t.}\; & b_{it}+\tau V_{it}+\sum_{j\in N}J_{ijt}v_{ijt}\\ & \;\;=\;\;b_{it-1}+\left(\sum_{l\in L}I_{lit}\right)u_{it}+\sum_{j\in N}\theta J_{jit}v_{jit}\\ & \;\forall \;i\in N\;\;t=1,\ldots ,T \end{array}$$

(2b) $$\begin{array}{cc} & \;\sum_{i\in N}I_{lit}u_{it}\;\;\leq \;\;c_{l}\\ & \;\;\forall \;l\in L\;\;t=1,\ldots ,T \end{array}$$

(2c) $$\begin{array}{cc} & \;\sum_{j\in N}\theta v_{jit}\;\;\leq \;\;\nu \\ & \;\;\forall \;i\in N\;\;t=1,\ldots ,T \end{array}$$

(2d) $$\begin{array}{cc} & \;\sum_{j\in N}v_{ijt}\;\;\leq \;\;\bar{\nu }\\ & \;\;\forall \;i\in N\;\;t=1,\ldots ,T \end{array}$$

(2e) $$\begin{array}{cc} & \;\sum_{i\in N}b_{i0}\;\;\leq \;\;\sum_{i\in N}b_{iT} \end{array}$$

(2f) $$\begin{array}{cc} & \;\sum_{l\in L}c_{l}\;\;\leq \;\;\gamma \end{array}$$

(2g) $$\begin{array}{cc} & \;0\;\;\leq \;\;u\;\;\leq \;\;\mu \end{array}$$

(2h) $$\begin{array}{cc} & \;\underset{‾}{\beta }\;\;\leq \;\;b\;\;\leq \;\;z \end{array}$$

(2i) c≥0

The formulations of problem (P2) are very similar to those of problem (P1), except we fix the (maximum) total charging capacity to γ. At the same time, we make the battery size (maximum battery level) a decision variable z instead of a constant β. Note that in problem (P2), we allow the charging capacity to be optimally allocated for the traveling and recharging plans of vehicles. This assumption follows the same idea as that in problem (P1), in that we assume drivers are smart enough to make informed travel and recharge plans and it avoids external uncertainty when related data are not available.

## 3 Results

To quantify the minimum requirement of a transportation system on the number of charging piles and capacity of vehicle batteries, we conducted numerical tests based on 24 h of Taxicab GPS data in Shenzhen, China [27]. We focused on a neighboring area of the Shenzhen Bao’an International Airport throughout the planning horizon, which is defined by longitude from 113.80 to 113.92, and latitude from 22.50 to 22.65. The dataset is thus filtered by longitude and latitude, which gave us 73 taxicabs that have stayed within the defined area throughout the planning horizon.

We aggregated the data records by partitioning the 24 h into 720 2-min periods. We then computed each taxicab’s mean latitude, longitude, and speed over each period. Additionally, we rounded the latitude and longitude to the nearest hundredth. Thus, we partitioned the region into geophysical grids by a 0.01 change in latitude and longitude. Therefore, the size of each grid is approximately 1 km by 1 km. We assumed that if two taxicabs are in the same geophysical grid during the same period, they can be arranged into the same platoon and transfer electricity to one another. Every geophysical grid has a potential location of a charging station. If a taxicab is parked (at zero speed) within a geophysical grid with a charging station, then it can refill its battery during that time period. Here, we chose 2-min time periods and 1-km by 1-km geophysical grids on the basis of the scale of our data instance, especially the number of vehicles. In practice, the actual number of vehicles in the system can be much larger than in the numerical tests. This allows for a much finer partition in time and space.

We consider the specifications of some popular electric vehicle models for parameter selection. Based on Table 2, we consider μ=2 (additional km per 2 min) for the scenario of regular 220 V charging. We consider multiple values of $\bar{\beta }$ near 400 km (because the actual mileages of electric cars are usually lower than those listed by carmakers, and decay over time as well), and we consider a reserved battery range $\underset{‾}{\beta }$ of 160 km (100 miles). We choose $\nu =\bar{\nu }=2$. That is, the maximum V2V charging rate should not exceed the regular 220 V charging rate from stations. Furthermore, we consider θ=0 for the scenario of no V2V charging, and we consider θ∈{0.50,0.75} for different scenarios of V2V charging. For example, θ=0.50 may represent that the V2V charging technology is in an early-stage of development, whereas θ=0.75 may represent that the technology is mature.

Remark 2 As the model input, task networks can have very different vehicle routes, itineraries, network structures, and system scales. Model (P1) and (P2) can take all kinds of task networks, but the outputs are highly dependent on the properties of the inputs. In this paper, we chose the taxicabs data, which is the most relevant and accessible to us. While taxicabs, ride-sharing vehicles, and private vehicles vary in their travel pattern, they are all serving the overall demand in the city with a similar capacity (vehicle size). Thus, we believe that our results generalize to the other two types of vehicles as well. That is, our results provide good estimations of the battery capacity and the number of charging piles needed to support a certain number of small electric vehicles within a city.

Remark 3 We provide the following justification for choosing a reserved battery range of 160 km: In case of an emergency, a car should be able to get to any place within the same city immediately without additional charging. The max radius of big cities around the world is usually the 1-h travel distance of the most commonly used transportation service (cars), which is approximately 80 km (or 50 miles). Thus we chose 160 km (or 100 miles) as the reserved battery range, which allows a car to travel from one end of a city to the other.

A reserved battery range of 160 km may seem very conservative. However, battery life decays over time. Since V2V charging introduces more charging and discharging (as electricity is passed along vehicles before being consumed), the vehicles’ battery life may decay even faster. Thus it is necessary to assume a sufficient reserved battery range such that the system can operate over time.

**Table 2:** **Specifications**[28], [29], [30]**of five of the most popular electric vehicle models in Shenzhen, from two of the largest EV manufacturers, ranked by total sales in China**[31].

Model	Max range	Battery	Typical charging speed
Tesla Model Y	330 miles	75 kWh	54 to 162 miles in 15 min (fast)
(2022)	or 531 km		8.5 to 10 h to full (220 V)
Tesla Model 3	358 miles	82 kWh	58 to 175 miles in 15 min (fast)
(2022)	or 576 km		8.5 to 10 h to full (220 V)
BYD Qin	249 miles	53 kWh	30 min to 80% (fast charging)
(2022)	or 401 km		8 to 9 h to full (220 V)
BYD Han	376 miles	77 kWh	25 min to 80% (fast charging)
(2022)	or 605 km		8 to 9 h to full (220 V)
BYD Tang	351 miles	83 kWh	30 min to 80% (fast charging)
(2022)	or 565 km		8 to 9 h to full (220 V)

**Fig. 3:**
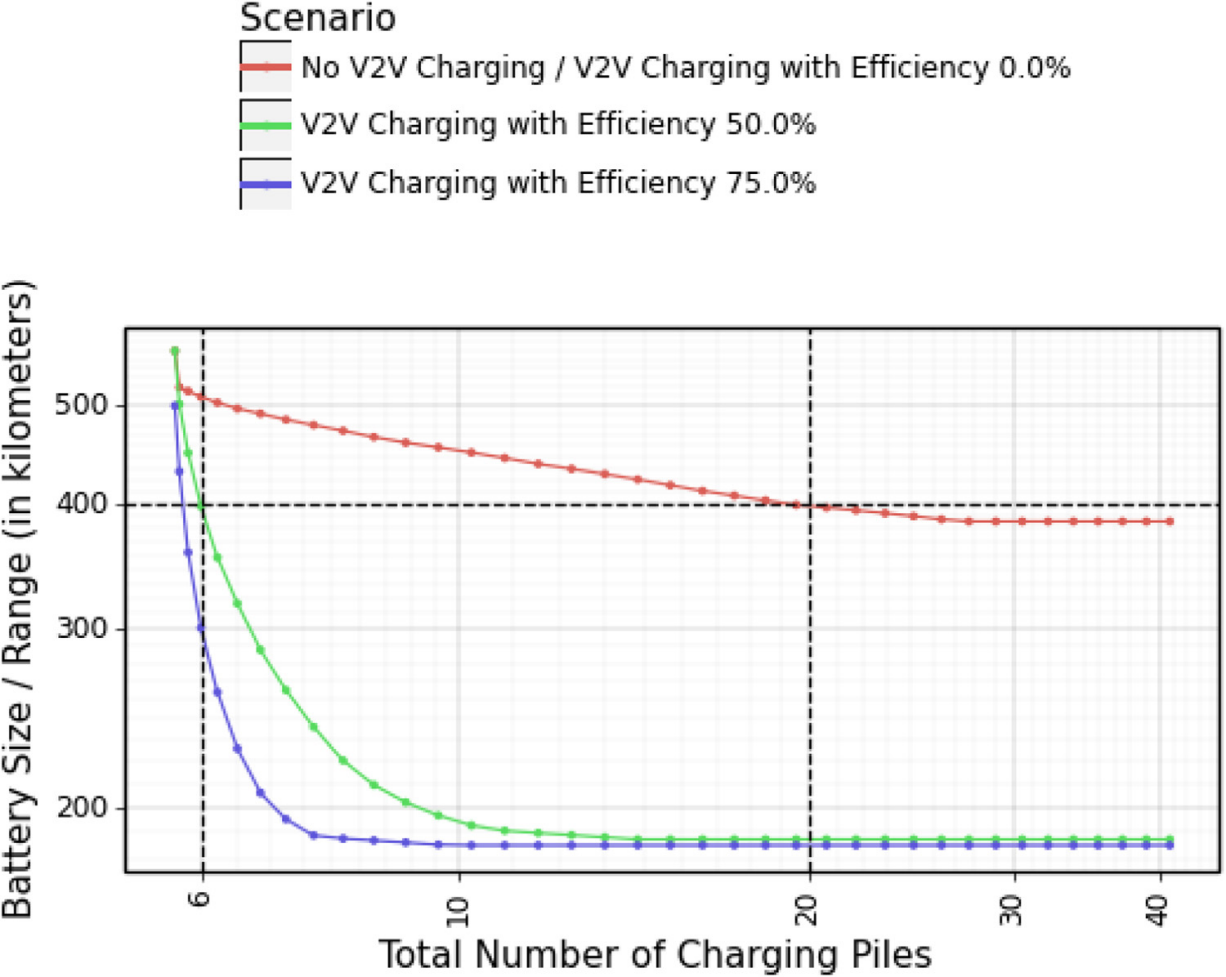
**The minimal battery size under different values of**γ**(total charging capacity) and**θ**(charging efficiency).** We use μ=2 (additional kilometers per 2 min) for the scenario of regular 220 V charging by piles.

Our results are summarized in Fig. 3. The results can be interpreted as follow:

*Less charging piles, higher utility* Assume that the vehicles have a battery size of 400 km. According to Fig. 3, the area needs 20 charging piles without V2V charging (i.e. the total charging capacity of all stations, γ, is 40 km per 2 min; while the capacity of each individual charging pile, μ, is 2 km per 2 min). When V2V charging with 50% efficiency is available, this number reduces to 6. (Note that the number 20 or 6 are not the *number* of charging stations, but the total *capacity* we can allocate across stations.) The second number is consistent with the situation in Shenzhen: In 2020, there were 362,800 electric vehicles and approximately 30,000 public charging piles in Shenzhen [32], which is 6.04 charging piles for every 73 electric vehicles. In other words, the current number of charging piles can be enough with even an elementary-level V2V charging technology. Without V2V charging, however, we will need at least 300% more charging piles to allow flexible traveling plans. (Note that the vehicles need to complete a similar travel plan under all scenarios, which means using V2V charging triples the utilization of charging piles.) Thus, while vehicles need more charging piles for more flexibility in travel, adopting V2V charging can significantly reduce the need for charging piles while preserving flexibility.

*A solution to range anxiety* If we have 6 charging piles for the 73 vehicles, the battery size can reduce to 300 km when V2V charging with 75% efficiency is available. When we increase the number of piles to 10, the battery size goes below 200 km. This result is robust to changes in V2V charging efficiency. In contrast, the battery size stays above 380 km without V2V charging. Thus, V2V charging can also be vital in addressing the issue of large vehicle batteries. In addition, Fig. 3 shows that the battery size is not sensitive to changes in charging capacity without V2V charging. This fact also hints that unless we adopt V2V charging technologies, building more charging piles will not ease range anxiety.

Remark 4 In Fig. 3, the two curves with V2V charging have high slopes towards the left. Thus, the required numbers of charging piles in those two scenarios are not sensitive to the change in the maximum battery size and the reserved battery range. Without V2V charging, however, the required number of charging piles can change dramatically as the choice of maximum battery size or reserved battery level changes. This could be a potential concern in interpreting our results. (Intuitively, a lower reserved battery range shifts the curves in Fig. 3 down. However, the actual impact on the required number of charging piles comes from the maximum battery level. For example, if we reduce the reserved battery range from 160 km to 100 km and then increase the maximum battery level from 400 km to 460 km, the required number of charging piles remains unchanged.)

In fact, the value of our results comes from this sensitivity itself. Without V2V charging, we have to choose from two options: to put a larger battery into every single car, or to install a lot more charging piles. Both options are very costly. If we take the decay of battery level into account, it can be even harder to make such a decision. V2V charging, even with only 50% efficiency, can take us out of this dilemma.

Remark 5 The total savings in charging pile installation and battery manufacturing can be understood as the “break-even point” in deploying the V2V charging technology. If the cost of installing V2V charging devices to all cars (as well as other additional costs, e.g., from potentially faster decay in battery life) is lower than installing more charging piles and making larger batteries, then it is profitable to deploy V2V charging. This is an approach we can use to predict the adoption of the V2V charging technology as it matures.

## 4 Discussion

V2V charging has the potential to address both range anxiety and the shortage of charging stations, and ease the heterogeneity of vehicle charging in time and space (e.g., some vehicles may drive for long periods and have little time to be charged by stationary stations, while others may frequently stop at locations without charging stations). Specifically, V2V charging (1) allows vehicles to be charged not only at a small number of charging stations, but also by a large number of other vehicles, (2) allows vehicles that drive for short distances and have long periods of time for charging to charge those driving for longer distances, (3) enable vehicles that have access to charging stations to charge those that have little access to them, and (4) save charging time by pairing two vehicles with overlapping routes for energy transmission.

While V2V wireless charging has the potential to become a game-changer, the maturity of the technology will likely be gradual, similar to that of electric vehicles. Therefore, this paper does not suggest halting the deployment of charging stations. Instead, we propose that researchers and practitioners consider the development of V2V charging and take a more robust approach to planning the deployment of charging station networks. In addition, we see several limitations in our formulation and model, which we list below to motivate future research:

*Economy of scale and change in vehicle weight* Two positive feedback effects are not modeled in this paper. First, V2V charging benefits from the economy of scale. With more vehicles in the system, it becomes easier for us to make good charging platoons. On the other hand, more vehicles will also join V2V charging as the system performance improves. Second, V2V charging allows electric vehicles to equip smaller batteries. As vehicles become more lightweight and consume less electricity [12], the overall requirement in charging capacity decreases. If the number of charging piles stays constant, the system will allow even smaller batteries. Thus, the benefit V2V charging brings to transportation systems can be even more significant than we analyzed. The resulting benefit to energy consumption, climate, and safety need to be further examined.

*Impact on battery life* Battery life will likely be impacted by V2V charging technology. The exact impact of a specific charging strategy on battery life is hard to quantify and is still under active research. However, it is widely believed that more frequent charging and discharging leads to faster decay in battery life. V2V charging introduces more charging and discharging as electricity is passed along multiple vehicles before being consumed. Our response to this possibility is to choose a very conservative reserved battery range. Yet it remains to be studied how much additional cost can incur from the change in battery life after the introduction of V2V charging and how this change compares to the reduction of cost from smaller batteries and fewer charging piles.

*Detours and dropping tasks* In this paper, we assume that all tasks need to be completed. We explained in Remark 1 why such a rigid assumption is made. However, there are special cases where this assumption needs to be relaxed. One possibility is that the data is small but contains many outliers (e.g., vehicle routes that are very long and do not overlap with any other routes). Our models have the flexibility to take a few outliers by adding more charging piles specifically for them. When the number of vehicles is large, such a change can be neglectable. However, the input data may have special structures and patterns, so the model must be tailored.

*Generalization to inter-city scenarios* While we focused on the intra-city scenario, our models and methods can be applied to other scenarios as long as input data is available. For example, the inter-city scenarios are also considered by many researchers and could be a good direction to extend our research. Intuitively, without V2V charging, the battery capacity is highly dependent on the maximum distance between origins and destinations. Long-distance travel will require many charging piles to be installed at the origins. With V2V charging, however, vehicles that travel long distances can get recharged en route from those that travel shorter distances. Since inter-city travels are long and highly overlapped, the benefit of V2V charging to the system can be more significant than under the intra-city scenario.

In addition, we list new research opportunities that may emerge from the business model shifts we envision:

*In-motion charging service* We assumed that all vehicles must strictly follow their original schedule extracted from GPS data in our models. However, allowing vehicles to take detours and facilitate better platooning for V2V charging sounds much more practical. For example, vehicles in a deficit of electricity may detour to recharge from other vehicles. When vehicles can purchase energy from other vehicles, a vehicle with a surplus of electricity may also detour to profit from charging other vehicles. Thus, service providers may design vehicles with huge batteries dedicated to charging other vehicles. A vehicle can then hail a service vehicle and charge it in motion, just the current ride-hailing service. This “in-motion charging service” can be essential to the existing on-demand vehicle charging service, which only services parked vehicles. Such systems’ optimal pricing and operation and their effect on congestion and emissions can also be valuable operations research topics.

*Standardization of vehicle batteries* V2V charging allows vehicle batteries to become smaller, which may lead to a standardization of vehicle batteries. Currently, the ability to produce large batteries is a core part of a carmaker’s competitiveness. With V2V charging, however, carmakers may no longer need to compete for making larger and larger batteries. Instead, they can focus on automotive technologies and rely on battery producers to supply standardized batteries. In addition, a car battery needs to be tailored specifically for a vehicle model in shape and structure to achieve its maximum capacity. However, when the battery can be smaller, its shape and structure will be more likely driven by manufacturing cost and thus become more standardized. The optimal sizing of batteries and passenger vehicles thus needs attention from researchers. Stepping further, the shift in the business model may facilitate the emergence of battery-swap services. This “battery-swap” solution should also be considered as an alternative to installing charging piles in future research.

*Modeling of energy flow in transportation research* With flexible energy transmission among vehicles, the energy management paradigm will shift from individual-based to group-based. As we have pointed out in Section 1, such a management paradigm can be very similar to that of cloud storage and computing, in which the resources can transmit among computers. In this paper, we considered the energy flow independent of the passenger flow. It is more challenging but exciting to view them as co-existing dimensions and to model their interdependence in future research on transportation systems.

## 5 Conclusion

We have demonstrated that V2V charging can be a game-changer in resolving both range anxiety and the shortage of charging piles. It eases the heterogeneity of charging needs in time and space. The results of our case study show that once V2V charging technologies with an efficiency of 50% are available, more than 2/3 of the charging piles investment would be wasted. Additionally, if the efficiency of V2V charging increases to 75%, we can easily reduce the battery capacity of vehicles to 200 km, reducing production costs and improving energy efficiency. These results may fundamentally change our path toward transportation electrification.

## Declaration of competing interest

The authors declare that they have no conflicts of interest in this work.

## Appendix A Supplementary materials

Supplementary Data S1 Supplementary Raw Research Data. This is open data under the CC BY license http://creativecommons.org/licenses/by/4.0/ Supplementary Data S1

Supplementary Data S2 Supplementary Raw Research Data. This is open data under the CC BY license http://creativecommons.org/licenses/by/4.0/ Supplementary Data S2
