# Supplementary material for: Are more charging piles imperative to future electrified transportation system?
Source: Fundam Res. 2022 Dec 24;4(5):1009–16. doi: 10.1016/j.fmre.2022.12.006 (PMC11489499; doi:10.1016/j.fmre.2022.12.006)
Supplement: Supplementary Data S1 — Supplementary Raw Research Data. This is open data under the CC BY license http://creativecommons.org/licenses/by/4.0/ [file mmc1.pdf]

Supplementary Materials for

An Alternative Pathway Towards Transportation Electrification via Vehicle-to-Vehicle Charging

Xiaobo Qu,[1*] Hongzhang Shao,[2] Shuaian Wang[3*]

[1]School of Vehicle and Mobility, Tsinghua University, Beijing, 100084 China
[2]School of Industrial and Systems Engineering, Georgia Institute of Technology, Atlanta, GA 30332, USA
[3]School of Mathematics and Applied Statistics, University of Wollongong, NSW 2522, Australia

[*]Corresponding authors: Xiaobo Qu (xiaobo@tsinghua.edu.cn), Shuaian Wang (wangshuaian@gmail.com)

September 5, 2022

Materials and Methods

Consider a region with N gasoline vehicles, and let $\mathcal{N}$ denote the set of all vehicles. Latitude, longitude, and speed data are collected for these vehicles over T time periods with an equal length τ. Suppose that the vehicles are to be replaced by electric vehicles. Using the data, we aim to estimate the minimal costs and requirements, such as the installation cost of new charging stations and the size of vehicle batteries, for this replacement to be possible. We also aim to determine whether V2V charging can reduce costs and requirements.

We formulate optimization models to find the minimal costs and requirements to ensure that every electric vehicle can complete its travel plan as specified by the dataset. Specifically, vehicles consume electricity when moving but can refill their batteries at a charging station. The optimization models allocate resources to ensure that the battery levels of all vehicles stay above a pre-determined safety level $\underline{\beta}$ throughout the planning horizon. As a convention, we use lowercase English letters for decision variables and indices, uppercase English letters for input data, and Greek letters for constant parameters. We assume that all vehicles have the same physical properties (i.e., they are identical in every aspect, including battery capacity, battery consumption rate, and maximum charging rate). Let V_{it} denote the average velocity of vehicle i during time period t for each $i \in \mathcal{N}$ and $t \in \{1, \ldots, T\}$. Let b_{it} denote the battery level of vehicle i at the end of time period t, and let b_{i0} denote the initial battery level of vehicle i (for convenience, we use the index $t \in \{1, \ldots, T\}$ to refer to both the time period and the end of that time period). Here, we measure battery level by the distance a vehicle can travel without having to stop to charge. Thus, each vehicle i consumes τV_{it} battery power during time period t. In other words, the energy consumption of a vehicle is proportional to its travel distance (or, equivalently, its average speed during a time period). If a vehicle is not traveling, it does not consume battery power.

Minimal Installation Cost of Charging Stations

We measure the installation cost by the total charging capacity of all charging stations instead of the integral number of charging piles for computational tractability. We assume that there are infinite positions at each charging station, such that all vehicles in the station can charge from it, as long as the total charging rate does not exceed its capacity. Thus, we characterize the charging capacity as the total amount of battery power vehicles can refill from the stations

within each time period. Let $\mathcal{L}$ denote the set of potential locations of charging stations. Let c_l denote the charging capacity of station $l \in \mathcal{L}$. For each $l \in \mathcal{L}$ and $i \in \mathcal{N}$, let $I_{lit} \in \{0, 1\}$ denote the indicator that takes value 1 if vehicle i can recharge itself at location l during time period t, and 0 otherwise. Here both $\mathcal{L}$ and $I := (I_{lit} : \ l \in \mathcal{L}, \ i \in \mathcal{N}, \ t \in \{1, \ldots, T\})$ are inputs that can be obtained from the data by applying certain rules. For example, $\mathcal{L}$ can be the set of all locations at which a vehicle has parked. If external data are available, $\mathcal{L}$ can also be the set of all parking lots, gas stations, and residential areas within the region. Note that each vehicle can charge from at most one station during each time period. Thus, we need $\sum_{l \in \mathcal{L}} I_{lit} \leq 1$ for every $i \in \mathcal{N}$ and $t \in \{1, \ldots, T\}$.

Vehicles refill batteries from charging stations. For each time period $t \in \{1, \ldots, T\}$ and each vehicle $i \in \mathcal{N}$, we let u_{it} denote the battery power recharged to vehicle i during time period t (the location of the charging station is indicated by I_{lit}). Let μ denote the maximum charging rate of vehicles (i.e., maximum battery power a vehicle can refill within a time period) from stations. That is, $u_{it} \leq \mu$ for any $i \in \mathcal{N}$ and $t \in \{1, \ldots, T\}$. In addition, let $\overline{\beta}$ denote the battery size (maximum battery level) of vehicles. That is, $b_{it} \leq \overline{\beta}$ for any $i \in \mathcal{N}$ and $t \in \{0, \ldots, T\}$.

Vehicles can also transfer electricity among one another. For each time period $t \in \{1, \ldots, T\}$ and each pair of vehicles $i, j \in \mathcal{N}$, we let v_{ijt} denote the battery power a vehicle i consumes to recharge the battery of vehicle j during time t, and $\bar{I}_{ijt} \in \{0, 1\}$ be the indicator that takes value 1 if such a recharge can happen (e.g. vehicle i and j can be arranged into the same platoon) during time period t, and 0 otherwise. We let $\theta \in [0, 1]$ denote the transferring efficiency. If vehicle i consumes battery power of v_{ijt} to recharge the battery of vehicle j during time period t, then vehicle j restores the battery level of θv_{ijt}. Thus, we can *disable* V2V charging by setting $\theta = 0$. Additionally, let ν denote the maximum (receiving) charging rate vehicles can receive

from other vehicles. That is, for any $i \in \mathcal{N}$ and $t \in \{1, \ldots, T\}$, we need $\sum_{j\in\mathcal{N}} \theta v_{jit} \leq \nu$. Similarly, let $\bar{\nu}$ denote the maximum (giving) charging rate vehicles can provide for other vehicles. That is, for any $i \in \mathcal{N}$ and $t \in \{1, \ldots, T\}$, we need $\sum_{j\in\mathcal{N}} v_{ijt} \leq \bar{\nu}$.

We can find the minimum total charging capacity of all stations by solving the following model:

$$\min_{b,\, c,\, u,\, v} \quad \sum_{l\in\mathcal{L}} c_l \tag{P$_1$}$$

$$\text{s.t.} \quad b_{it} + \tau V_{it} + \sum_{j\in\mathcal{N}} \bar{I}_{ijt} v_{ijt}$$

$$= b_{it-1} + \left(\sum_{l\in\mathcal{L}} I_{lit}\right) u_{it} + \sum_{j\in\mathcal{N}} \theta \bar{I}_{jit} v_{jit} \qquad \forall\, i \in \mathcal{N}\,,\ t = 1, \cdots, T \tag{1a}$$

$$\sum_{i\in\mathcal{N}} I_{lit} u_{it} \leq c_l \qquad \forall\, l \in \mathcal{L}\,,\ t = 1, \cdots, T \tag{1b}$$

$$\sum_{j\in\mathcal{N}} \theta v_{jit} \leq \nu \qquad \forall\, i \in \mathcal{N}\,,\ t = 1, \cdots, T \tag{1c}$$

$$\sum_{j\in\mathcal{N}} v_{ijt} \leq \bar{\nu} \qquad \forall\, i \in \mathcal{N}\,,\ t = 1, \cdots, T \tag{1d}$$

$$\sum_{i\in\mathcal{N}} b_{i0} \leq \sum_{i\in\mathcal{N}} b_{iT} \tag{1e}$$

$$0 \leq u \leq \mu \tag{1f}$$

$$\underline{\beta} \leq b \leq \overline{\beta} \tag{1g}$$

$$v \geq 0 \tag{1h}$$

$$c \geq 0. \tag{1i}$$

In (P$_1$), constraint (1a) ensures that vehicles follow the rules of consuming electricity with regards to moving and refilling electricity from charging stations (while parked) or other vehicles. Constraint (1b) then checks that the total charging rate to all vehicles from each station does not exceed its capacity (recall that $\sum_{l\in\mathcal{L}} I_{lit} \leq 1$). Note that in (P$_1$), battery levels, as well as initial battery levels, are decision variables. The idea here is that we assume that drivers are able to

determine the best travel and recharge plans for themselves. We then use constraint (1e) to prevent "overfitting" and free ourselves from the impact of the conditions of initial battery levels. Intuitively, drivers should still be capable of future travel at the end of the planning horizon. Thus, the total ending battery level should be greater or equal to the total initial battery level.

Remark 1 *As a network flow problem, the value of variable v forms a tree structure at optimality. Specifically, the transfer of electricity should not contain circles (otherwise, electricity is wasted). Here (P_1) allows electricity to be transferred between multiple vehicles simultaneously (i.e., a vehicle can receive electricity from multiple givers and can transmit electricity to multiple receivers during the same time period). In cases in which electricity can only be transferred between vehicles one pair at a time, we can perform "time-division multiplexing" by dividing each time period into smaller sub-periods and then work with different charging pairs during different sub-periods. Thus, the solution from (P_1) will still be valid.*

Remark 2 *When data are available, it is more realistic to assume that there are already charging stations installed in the region. In this case, we can add constraints $c_l \geq C_l$, $l \in \mathcal{L}$ to (P_1), where for each $l \in \mathcal{L}$, C_l is the capacity that is already installed at location l. We then simply need to subtract $\sum_{l \in \mathcal{L}} C_l$ from the objective value, because this existing capacity is already installed.*

Minimum Size of Vehicle Batteries

The values of parameters μ, ν, $\bar{\nu}$, θ, $\underline{\beta}$, and $\overline{\beta}$ may have strong impacts on the solution of (P_1) and (P_1). Whereas charging rates μ, ν, $\bar{\nu}$ and charging efficiency θ are relatively exogenous in practice, battery size $\overline{\beta}$ can be controlled. However, battery accounts for a large portion

of the cost of producing electric vehicles, and reducing the battery size can be profitable for vehicle manufacturers. Thus, it is necessary to establish the minimum size of vehicle batteries, considering the travel plans of vehicles and the capacities of charging stations.

Let z denote the battery size of vehicles. Let γ denote the total charging capacity. Then the minimum size of vehicle batteries can be found by solving the following model:

$$
\begin{aligned}
\min_{b,\, c,\, u,\, v,\, z} \quad & z && && (\mathsf{P}_2)\\
\text{s.t.} \quad & b_{it} + \tau V_{it} + \sum_{j\in\mathcal{N}} \bar{I}_{ijt} v_{ijt} && && \\
& \quad = \; b_{it-1} + \left(\sum_{l\in\mathcal{L}} I_{lit}\right) u_{it} + \sum_{j\in\mathcal{N}} \theta \bar{I}_{jit} v_{jit} && \forall\, i \in \mathcal{N},\ t = 1,\cdots,T && (2a)\\
& \sum_{i\in\mathcal{N}} I_{lit} u_{it} \;\le\; c_l && \forall\, l \in \mathcal{L},\ t = 1,\cdots,T && (2b)\\
& \sum_{j\in\mathcal{N}} \theta v_{jit} \;\le\; \nu && \forall\, i \in \mathcal{N},\ t = 1,\cdots,T && (2c)\\
& \sum_{j\in\mathcal{N}} v_{ijt} \;\le\; \bar{\nu} && \forall\, i \in \mathcal{N},\ t = 1,\cdots,T && (2d)\\
& \sum_{i\in\mathcal{N}} b_{i0} \;\le\; \sum_{i\in\mathcal{N}} b_{iT} && && (2e)\\
& \sum_{l\in\mathcal{L}} c_l \;\le\; \gamma && && (2f)\\
& 0 \;\le\; u \;\le\; \mu && && (2g)\\
& \underline{\beta} \;\le\; b \;\le\; z && && (2h)\\
& c \;\ge\; 0. && && (2i)
\end{aligned}
$$

The formulations of (P_2) are very similar to those of (P_1), except we fix the (maximum) total charging capacity to γ. At the same time, we make the battery size (maximum battery level) a decision variable z instead of a constant β. Note that in (P_2), we allow the charging capacity to be optimally allocated for the traveling and recharging plans of vehicles. This assumption

follows the same idea as that in (P_1), in that we assume drivers are smart enough to make informed travel and recharge plans and it avoids external uncertainty when related data are not available.

Data

We conduct numerical tests based on 24 hours of taxicab GPS data in Shenzhen, China (*1*). We analyze the longitude, latitude, and speed data of 73 taxicabs within an area neighboring Shenzhen Bao'an International Airport throughout the planning horizon. The area is defined by longitude from 113.80 to 113.92, and latitude from 22.50 to 22.65. Sample trajectories of taxicabs from the original dataset and the filtered dataset are provided in Figure S1 and Figure S2 respectively.

We aggregated the data records by partitioning the 24 hours into 720 2-minute periods. We then compute each taxicab's mean latitude, longitude, and speed over each period. Additionally, we round the latitude and longitude to the nearest hundredth. Thus, we partition the region into geophysical grids by a 0.01 change in latitude and longitude. Therefore, the size of each grid is approximately 1 km by 1 km. We assume that if two taxicabs are in the same geophysical grid during the same period, they can be arranged into the same platoon and transfer electricity to one another. We also assume that every geophysical grid has a charging station. If a taxicab is parked (at zero speed) within a geophysical grid with a charging station, then it can refill its battery during that time period.

Remark 3 *We choose 2-minute time periods and 1-km by 1-km geophysical grids on the basis of the scale of our data instance, especially the number of vehicles. Figure S4 shows the histogram of vehicle count within each time-space grid. In practice, the actual number of vehicles*

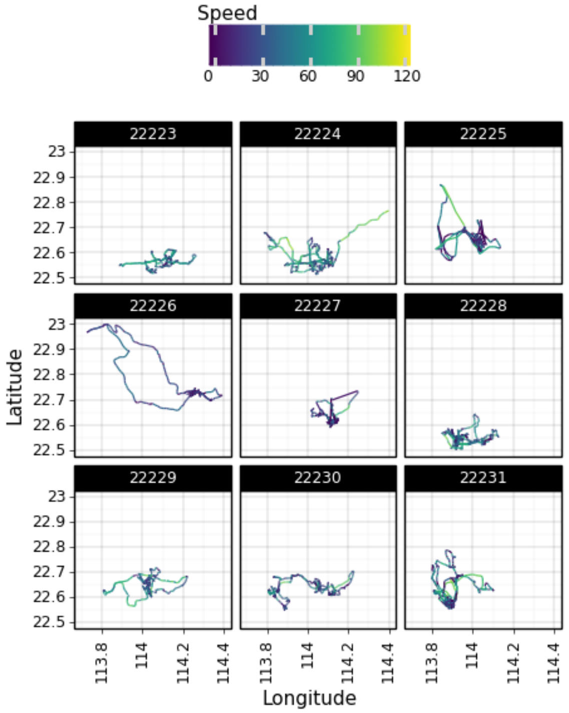

Figure S1: Sample trajectories of taxicabs from the original dataset over the 24-hour planning horizon. The five-digit numbers above the plots are identifiers of individual taxicabs.

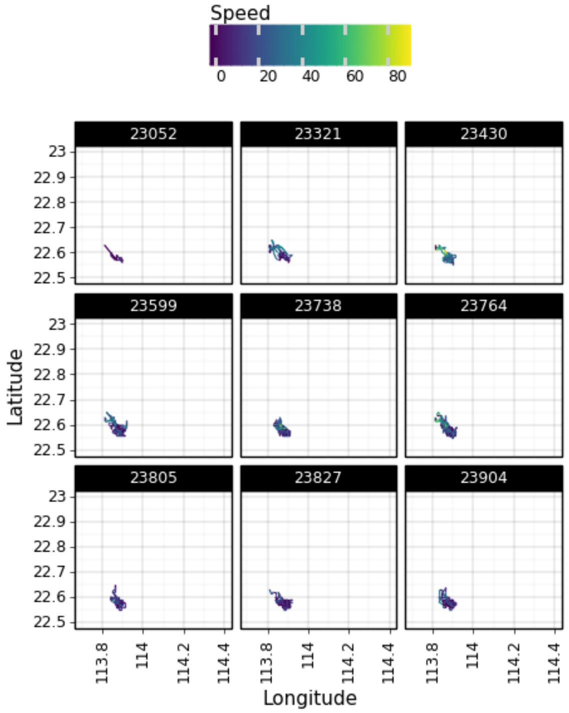

Figure S2: Sample trajectories of taxicabs from the filtered dataset over the 24-hour planning horizon. The five-digit numbers above the plots are identifiers of individual taxicabs.

in the system can be much larger than in the numerical tests. This allows for a much more detailed partition in time and space.

We consider the specifications of some popular electric vehicle models for parameter selection. Based on Table S1, we consider $\mu = 2$ (additional km per 2 minutes) for the scenario of regular 220V charging, and we consider $\mu = 20$ for the scenario of fast charging. We consider multiple values of $\overline{\beta}$ near 400 km (because the actual mileages of electric cars are usually lower than those listed by carmakers), and we consider a safe battery level $\underline{\beta}$ of 160 km (100

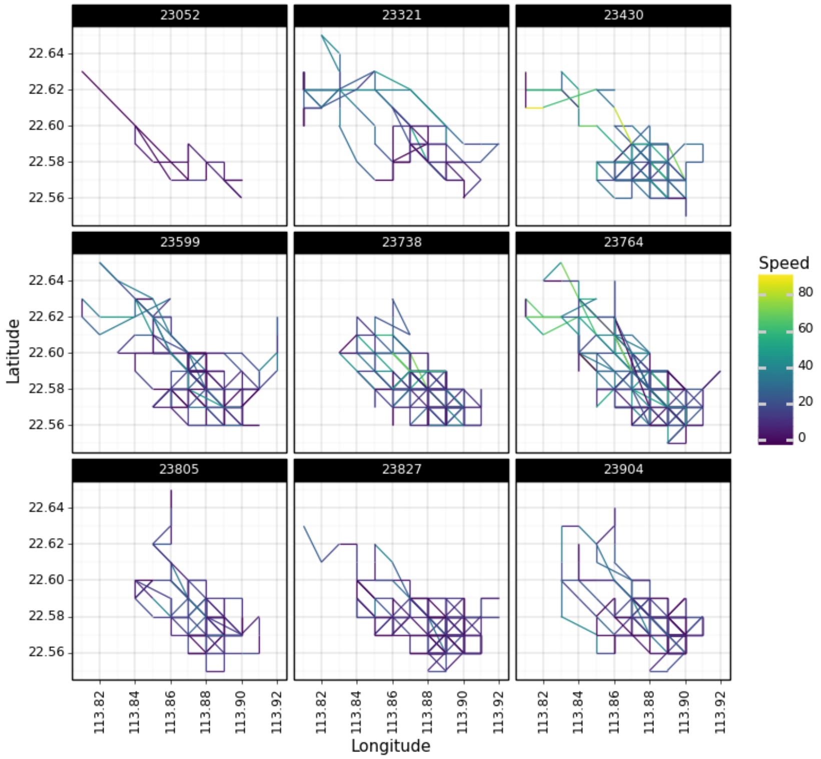

Figure S3: Sample trajectories of taxicabs from the aggregated dataset (zoomed). Note that we partitioned the region into geophysical grids.

miles). We choose $\nu = \bar{\nu} = 2$. That is, the maximum V2V charging rate should not exceed the regular 220V charging rate from stations. Furthermore, we consider $\theta = 0$ for the scenario of no V2V charging, and we consider $\theta \in \{0.50, 0.75\}$ for different scenarios of V2V charging. For example, $\theta = 0.50$ may represent that the V2V charging technology is in an early-stage of development, whereas $\theta = 0.75$ may represent that the technology is mature.

Figures S6 and S7 summarize the two sets of results. Discussions are presented in the main article.

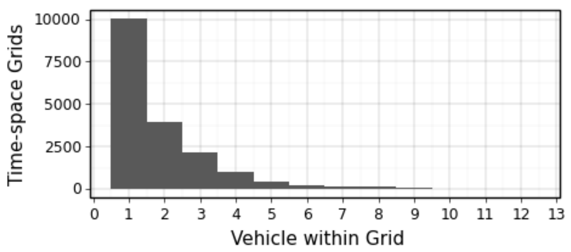

Figure S4: Counts of pairs of geophysical grids and time periods with different numbers of taxicabs in each. When there are two or more taxicabs in the same geophysical grid during the same time period, V2V charging can occur.

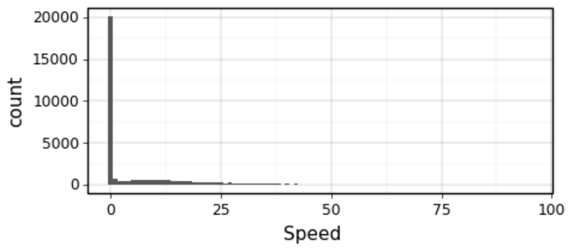

Figure S5: Histogram of speed for all taxicabs and all time periods. Note that most of the time, the speed of the taxicabs is below 30 km/hour. This justifies our aggregation of data by 2-minute time periods and 1-km by 1-km geophysical grids.

Model	Max Range	Typical Battery	Typical Charging Speed
Tesla Model Y (2022)	330 miles or 531 km	75 kWh	54 to 162 miles in 15 minutes (fast charging)<br>8.5 to 10 hour to full (220V)
Tesla Model 3 (2022)	358 miles or 576 km	82 kWh	58 to 175 miles in 15 minutes (fast charging)<br>8.5 to 10 hour to full (220V)
BYD Qin (2022)	249 miles or 401 km	53 kWh	30 minutes to 80% (fast charging)<br>8 to 9 hour to full (220V)
BYD Han (2022)	376 miles or 605 km	77 kWh	25 minutes to 80% (fast charging)<br>8 to 9 hour to full (220V)
BYD Tang (2022)	351 miles or 565 km	83 kWh	30 minutes to 80% (fast charging)<br>8 to 9 hour to full (220V)

Table S1: Specifications (*2*) (*3*) (*4*) of five of the most popular electric vehicle models in Shenzhen (*5*), from two of the largest EV manufacturers (ranked by total sales in China) (*5*).

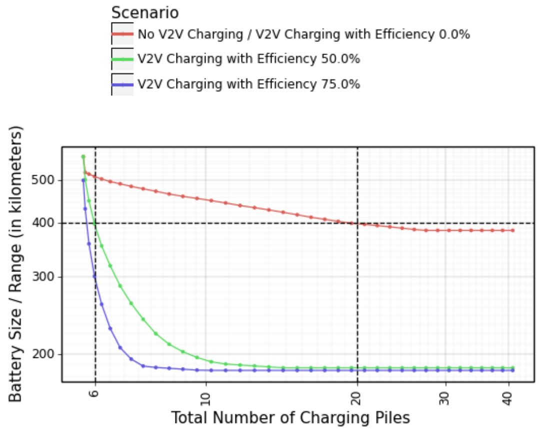

Figure S6: The minimal battery size, solved from (P_2), under different values of γ (total charging capacity) and θ (charging efficiency). We use $\mu = 2$ (additional kilometers per 2 minutes) for the scenario of regular 220V charging by piles.

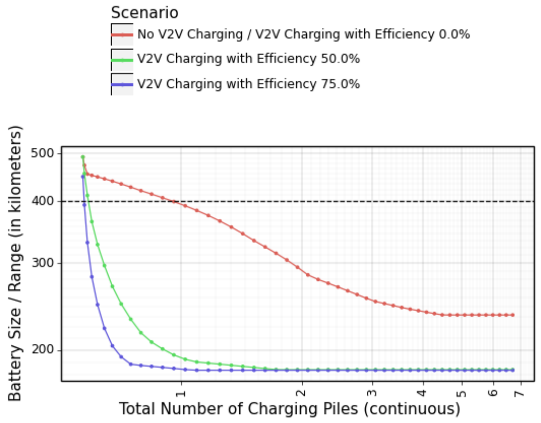

Figure S7: The minimal battery size, solved from (P_2), under different values of γ (total charging capacity) and θ (charging efficiency). We use $\mu = 20$ (additional kilometers per 2 minutes) for the scenario of fast charging by piles.

References

1. D. Zhang, J. Zhao, F. Zhang, T. He, *Proceedings of the ACM/IEEE Sixth International Conference on Cyber-Physical Systems* (2015), pp. 238–247.

2. BYD, Auto, https://en.byd.com/auto/. Online; accessed 22-Feb-2022.

3. Tesla, Model 3, https://www.tesla.com/model3/. Online; accessed 22-Feb-2022.

4. Tesla, Model y, https://www.tesla.com/modely/. Online; accessed 22-Feb-2022.

5. China Passenger Car Association (CPCA), Monthly report of new energy vehicle industry (2021-12), http://www.cpcaauto.com/newslist.php?types=bgzl&id=1059/. Online; accessed 22-Feb-2022.
