# Supplementary material for: Are more charging piles imperative to future electrified transportation system?
Source: Fundam Res. 2022 Dec 24;4(5):1009–16. doi: 10.1016/j.fmre.2022.12.006 (PMC11489499; doi:10.1016/j.fmre.2022.12.006)
Supplement: Supplementary Data S2 — Supplementary Raw Research Data. This is open data under the CC BY license http://creativecommons.org/licenses/by/4.0/ [file mmc2.pdf]

Data source: https://www.cs.rutgers.edu/~dz220/data.html

In [ ]:
```
# ---------- Prepare Data : Urban Data Release V2 (Taxi) ---------- #
# df = pd.read_csv("raw-taxi.csv")
# df.columns = ['CarID', 'Time', 'Longitude', 'Latitude', 'Occupied', 'Speed']
# df = df.sort_values(by = ['CarID', 'Time'], ascending = [True, True])
# df.to_csv('gps-data-shenzhen-taxi.csv')
```

Data Exploration

In [2]:
```
import numpy as np
import cvxpy as cp
import pandas as pd

from plotnine import *

cp.installed_solvers()
```

Out[2]:
```
['CPLEX', 'ECOS', 'ECOS_BB', 'GUROBI', 'OSQP', 'SCIPY', 'SCS']
```

In [3]:
```
# read data
df = pd.read_csv("gps-data-shenzhen-taxi.csv", index_col=0)
display(df.dtypes)
```

```
CarID          int64
Time          object
Longitude    float64
Latitude     float64
Occupied       int64
Speed          int64
dtype: object
```

In [4]:
```
print('Total number of records. : ', df.shape[0])
print('Total number of vehicles : ', len(df['CarID'].unique()))
```

```
Total number of records. :  46927854
Total number of vehicles :  14728
```

Below are samples of car paths. Note that we lost track of some cars occasionally (cars move to a different place with zero speed).

In [5]:
```
chart = (
    ggplot(df.loc[df['CarID'].isin([22223,22224,22225,22226,22227,22228,22229,22230,22231])])
    + geom_path(aes(x='Longitude', y='Latitude', color='Speed'))
    + facet_wrap('CarID', nrow=3)
    + theme_linedraw()
    + theme(legend_position='top')
    + xlim(113.7, 114.4) + ylim(22.5, 23.0)
    + theme(figure_size=(5, 5), legend_position='top')
    + theme(axis_text_x = element_text(angle=90))
)

chart.save("sample-trajectories.png")
chart
```

```
C:\Users\hshao33\AppData\Roaming\Python\Python38\site-packages\plotnine\ggplot.py:719: PlotnineWarning: Saving 5 x 5 in image.
C:\Users\hshao33\AppData\Roaming\Python\Python38\site-packages\plotnine\ggplot.py:722: PlotnineWarning: Filename: sample-trajectori
es.png
```

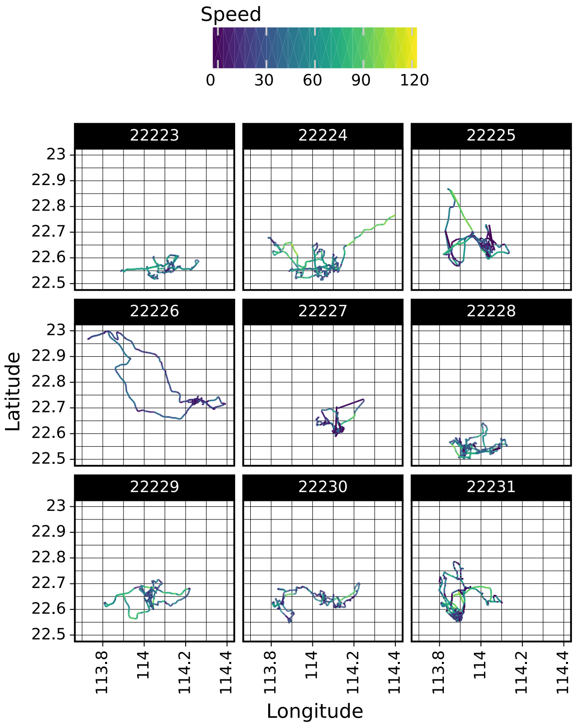

Out[5]: <ggplot: (145664952540)>

Discretize time and save results

In [6]:

```
df['T'] = (df['Time'].str[:2].astype(int)) * 60 + (df['Time'].str[3:5].astype(int))
df['T'] = df['T'] / 2
df['T'] = df['T'].round()
df['T'] = df['T'].astype(int)
```

In [7]:

```
df = df.groupby(by=['T', 'CarID']).mean().round(2)
df = df.reset_index()
df
```

Out[7]:

	T	CarID	Longitude	Latitude	Occupied	Speed
**0**	0	22223	114.15	22.56	1.0	66.55
**1**	0	22224	114.04	22.56	0.0	50.38
**2**	0	22226	114.28	22.72	0.0	0.00
**3**	0	22227	114.12	22.60	1.0	11.80
**4**	0	22228	113.98	22.54	1.0	61.86
**...**	...	...	...	...	...	...
**9166761**	720	36935	114.14	22.59	0.0	5.00
**9166762**	720	36937	114.08	22.54	0.0	0.00
**9166763**	720	36940	114.01	22.53	0.0	35.00
**9166764**	720	36944	114.11	22.54	1.0	12.50
**9166765**	720	36949	114.10	22.55	0.0	0.00

9166766 rows × 6 columns

In [8]:
```
df.to_csv("gps-data-shenzhen-taxi-agg.csv", index = False)
```

Data Filtering

In [9]:
```
import numpy as np
import cvxpy as cp
import pandas as pd

from plotnine import *

cp.installed_solvers()
```

Out[9]: ['CPLEX', 'ECOS', 'ECOS_BB', 'GUROBI', 'OSQP', 'SCIPY', 'SCS']

In [10]:
```
df = pd.read_csv("gps-data-shenzhen-taxi-agg.csv")
df
```

Out[10]:

	T	CarID	Longitude	Latitude	Occupied	Speed
**0**	0	22223	114.15	22.56	1.0	66.55
**1**	0	22224	114.04	22.56	0.0	50.38
**2**	0	22226	114.28	22.72	0.0	0.00
**3**	0	22227	114.12	22.60	1.0	11.80
**4**	0	22228	113.98	22.54	1.0	61.86
**...**	...	...	...	...	...	...
**9166761**	720	36935	114.14	22.59	0.0	5.00
**9166762**	720	36937	114.08	22.54	0.0	0.00
**9166763**	720	36940	114.01	22.53	0.0	35.00
**9166764**	720	36944	114.11	22.54	1.0	12.50
**9166765**	720	36949	114.10	22.55	0.0	0.00

9166766 rows × 6 columns

In [11]:
```
df_agg = df.groupby(by=['T', 'Longitude', 'Latitude']).count()
df_agg = df_agg.loc[df_agg['Occupied'] > 1]
df_agg.reset_index(inplace=True)
df_agg
```

Out[11]:

	T	Longitude	Latitude	CarID	Occupied	Speed
**0**	0	113.79	22.68	2	2	2
**1**	0	113.80	22.67	5	5	5
**2**	0	113.80	22.68	7	7	7
**3**	0	113.80	22.69	3	3	3
**4**	0	113.80	22.70	4	4	4
**...**	...	...	...	...	...	...
**410525**	720	114.30	22.59	3	3	3
**410526**	720	114.30	22.60	3	3	3
**410527**	720	114.30	22.72	2	2	2
**410528**	720	114.30	22.77	2	2	2
**410529**	720	114.34	22.69	2	2	2

410530 rows × 6 columns

In [12]:
```
df_agg['Speed'].hist(bins=200, range=(0,200))
```

Out[12]: <AxesSubplot:>

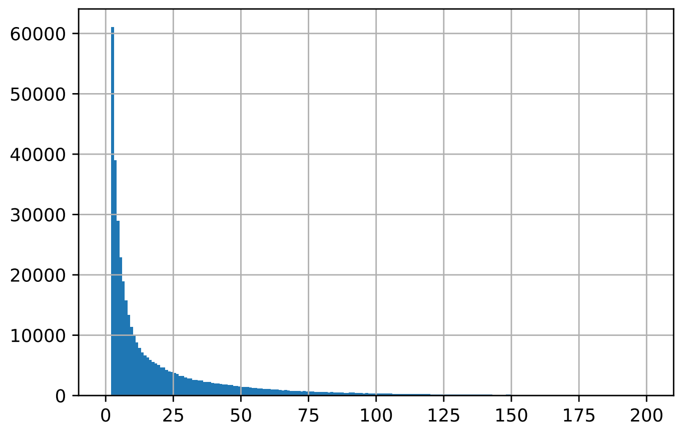

Number of cars in each pair time period and geo grid

In [13]:

```
df_agg = df.groupby(by=['T', 'CarID']).mean().round(2)
df_agg['Speed'].hist(bins=100, range=(0,100))
```

Out[13]: <AxesSubplot:>

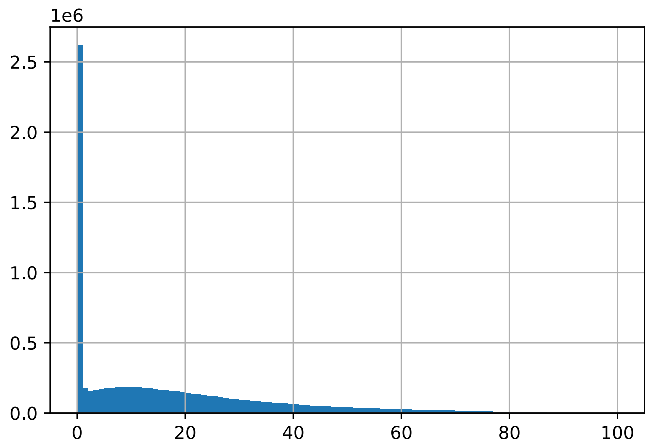

In [14]:

```
df_agg = df_agg.groupby(by=['T', 'Longitude', 'Latitude']).count()
df_agg = df_agg.reset_index()

print('Number of Geographic Grids : ', df_agg.groupby(by=['Longitude', 'Latitude']).count().shape[0])
print('Number of Time Periods     : ', df_agg.groupby(by=['T']).count().shape[0])
```

```
Number of Geographic Grids :  4735
Number of Time Periods     :  721
```

In [15]:

```
df_prep = df.groupby(by=['CarID']).agg({'Speed': ['min', 'max'],
                                        'Longitude': ['min', 'max'],
                                        'Latitude': ['min', 'max'],
                                        'T': 'count'})

df_prep['T']['count'].hist(bins=144, range=(0,721))
```

Out[15]: <AxesSubplot:>

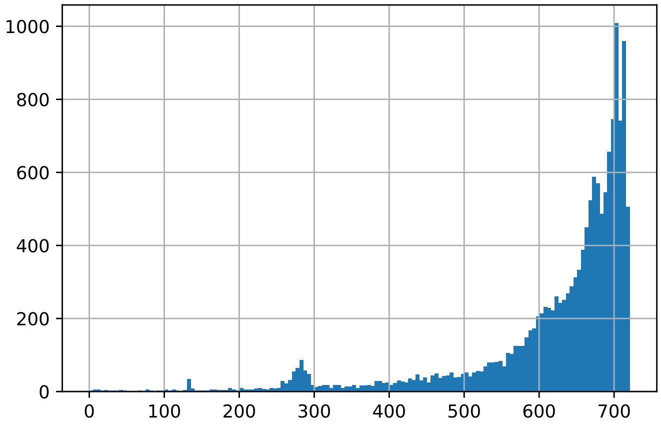

In [16]:
```
df_prep['Longitude']['min'].hist(bins=100, range=(113.51,114.50))
df_prep['Longitude']['max'].hist(bins=100, range=(113.51,114.50))
```

Out[16]: <AxesSubplot:>

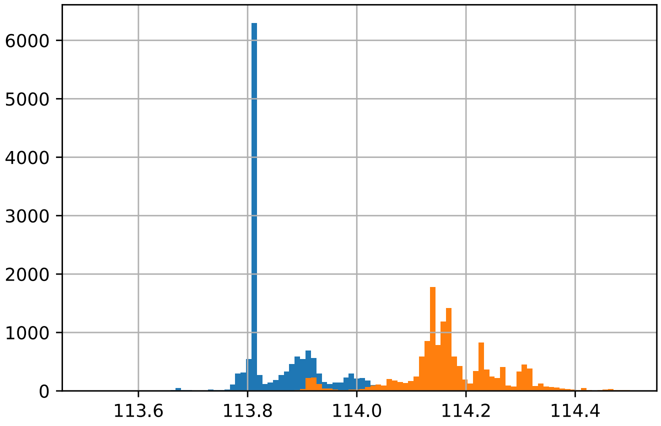

In [17]:
```
df_prep['Latitude']['min'].hist(bins=50, range=(22.31,22.80))
df_prep['Latitude']['max'].hist(bins=50, range=(22.31,22.80))
```

Out[17]: <AxesSubplot:>

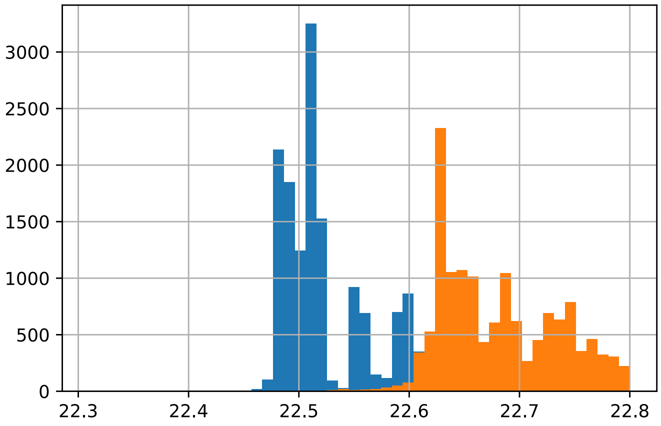

Filter data by number of records and range of latitude / longitude:

In [18]:

```
df_prep = df.groupby(by=['CarID']).agg({'Speed': ['min', 'max'],
                                        'Longitude': ['min', 'max'],
                                        'Latitude': ['min', 'max'],
                                        'T': 'count'})


# df_prep = df_prep[df_prep['T']['count'] >= 700]
# print('Number of cars left :', df_prep.shape[0])

df_prep = df_prep[df_prep['Longitude']['min'] >= 113.8]
print('Number of cars left :', df_prep.shape[0])

df_prep = df_prep[df_prep['Longitude']['max'] <= 113.92]
print('Number of cars left :', df_prep.shape[0])

df_prep = df_prep[df_prep['Latitude']['min'] >= 22.5]
print('Number of cars left :', df_prep.shape[0])

df_prep = df_prep[df_prep['Latitude']['max'] <= 22.65]
print('Number of cars left :', df_prep.shape[0])
```

```
Number of cars left : 13738
Number of cars left : 332
Number of cars left : 332
Number of cars left : 73
```

In [19]:

```
df_agg = df.loc[df['CarID'].isin(df_prep.index)].groupby(by=['T', 'Longitude', 'Latitude']).count()
df_agg.reset_index(inplace=True)
df_agg['Speed'].hist(bins=20, range=(0,20))
```

Out[19]: <AxesSubplot:>

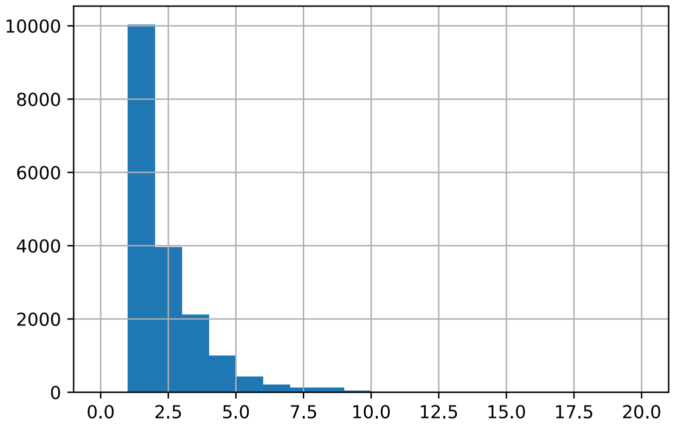

Arrange and save filtered data:

In [20]:

```
mydf = df[df['CarID'].isin(df_prep.index)]
mydf
```

Out[20]:

	T	CarID	Longitude	Latitude	Occupied	Speed
**325**	0	22596	113.92	22.52	1.0	0.0
**744**	0	23052	113.81	22.63	0.0	0.0
**983**	0	23321	113.89	22.59	0.0	0.0
**1081**	0	23430	113.89	22.59	1.0	9.0
**1236**	0	23599	113.88	22.59	0.0	14.0
**...**	...	...	...	...	...	...
**9163113**	720	24902	113.91	22.65	0.0	0.0
**9163636**	720	26600	113.85	22.58	0.0	0.0
**9163895**	720	27413	113.89	22.59	0.0	0.0
**9164262**	720	28669	113.89	22.59	0.0	10.0
**9165228**	720	31955	113.90	22.56	0.0	0.0

34460 rows × 6 columns

In [21]:

```
mydf['T'].hist(bins=72, range=(0,720))
```

Out[21]: <AxesSubplot:>

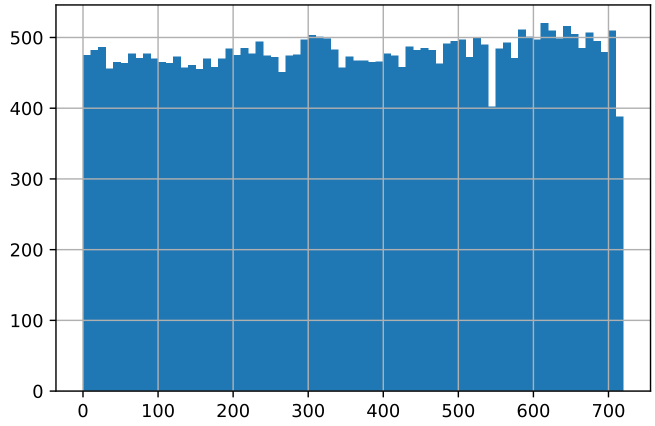

In [22]:

```
mydf.to_csv("gps-data-shenzhen-taxi-small-73.csv", index = False)
```

Data Visualizations

In [23]:

```
# read data
mydf = pd.read_csv("gps-data-shenzhen-taxi-small-73.csv")
display(mydf.dtypes)
```

```
T               int64
CarID           int64
Longitude     float64
Latitude      float64
Occupied      float64
Speed         float64
dtype: object
```

In [27]:

```
from mizani.transforms  import log_trans


print('Total number of records. : ', mydf.shape[0])
print('Total number of vehicles : ', len(mydf['CarID'].unique()))

chart = (
    ggplot(mydf)
    + geom_histogram(aes(x='Speed'), binwidth=1)
    + theme_linedraw()
    + theme(figure_size=(5, 2))
)

chart.save("histogram-speed-73-cars")
chart
```

```
Total number of records. :  34460
Total number of vehicles :  73
C:\Users\hshao33\AppData\Roaming\Python\Python38\site-packages\plotnine\ggplot.py:719: PlotnineWarning: Saving 5 x 2 in image.
C:\Users\hshao33\AppData\Roaming\Python\Python38\site-packages\plotnine\ggplot.py:722: PlotnineWarning: Filename: histogram-speed-7
3-cars
```

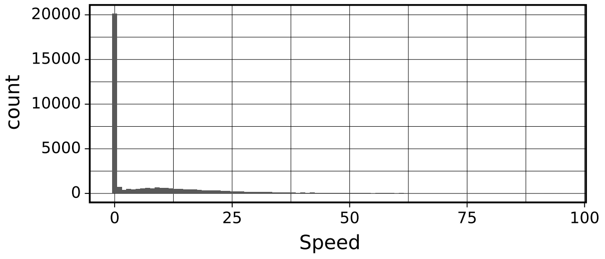

Out[27]: <ggplot: (145493256421)>

In [29]:

```
chart = (
    ggplot(mydf.loc[mydf['CarID'].isin(mydf['CarID'].unique()[1:10])])
    + geom_path(aes(x='Longitude', y='Latitude', color='Speed'))
    + facet_wrap('CarID', nrow=3)
    + theme_linedraw()
    + theme(legend_position='top')
    + xlim(113.7, 114.4) + ylim(22.5, 23.0)
    + theme(figure_size=(5, 5), legend_position='top')
    + theme(axis_text_x = element_text(angle=90))
)

chart.save("sample-trajectories-73-cars.png")
chart
```

```
C:\Users\hshao33\AppData\Roaming\Python\Python38\site-packages\plotnine\ggplot.py:719: PlotnineWarning: Saving 5 x 5 in image.
C:\Users\hshao33\AppData\Roaming\Python\Python38\site-packages\plotnine\ggplot.py:722: PlotnineWarning: Filename: sample-trajectori
es-73-cars.png
```

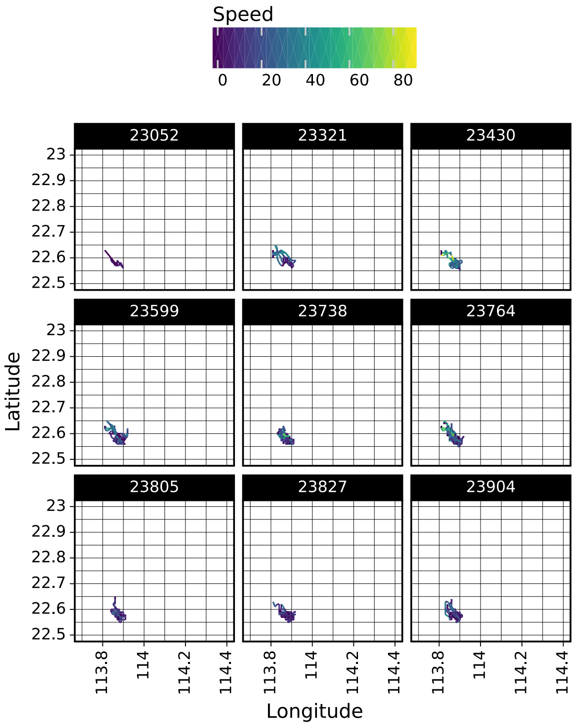

Out[29]: <ggplot: (145602656991)>

In [30]:

```
chart = (
    ggplot(mydf.loc[mydf['CarID'].isin(mydf['CarID'].unique()[1:10])])
    + geom_path(aes(x='Longitude', y='Latitude', color='Speed'))
    + facet_wrap('CarID', nrow=3)
    + theme_linedraw()
    + theme(legend_position='top')
    + theme(figure_size=(8, 8), legend_position='right')
    + theme(axis_text_x = element_text(angle=90))
    + scale_x_continuous(breaks=np.arange(113.8, 114.0, 0.02))
    + scale_y_continuous(breaks=np.arange(22.5, 22.7, 0.02))
)

chart.save("sample-trajectories-zoomed.png")
chart
```

```
C:\Users\hshao33\AppData\Roaming\Python\Python38\site-packages\plotnine\ggplot.py:719: PlotnineWarning: Saving 8 x 8 in image.
C:\Users\hshao33\AppData\Roaming\Python\Python38\site-packages\plotnine\ggplot.py:722: PlotnineWarning: Filename: sample-trajectori
es-zoomed.png
```

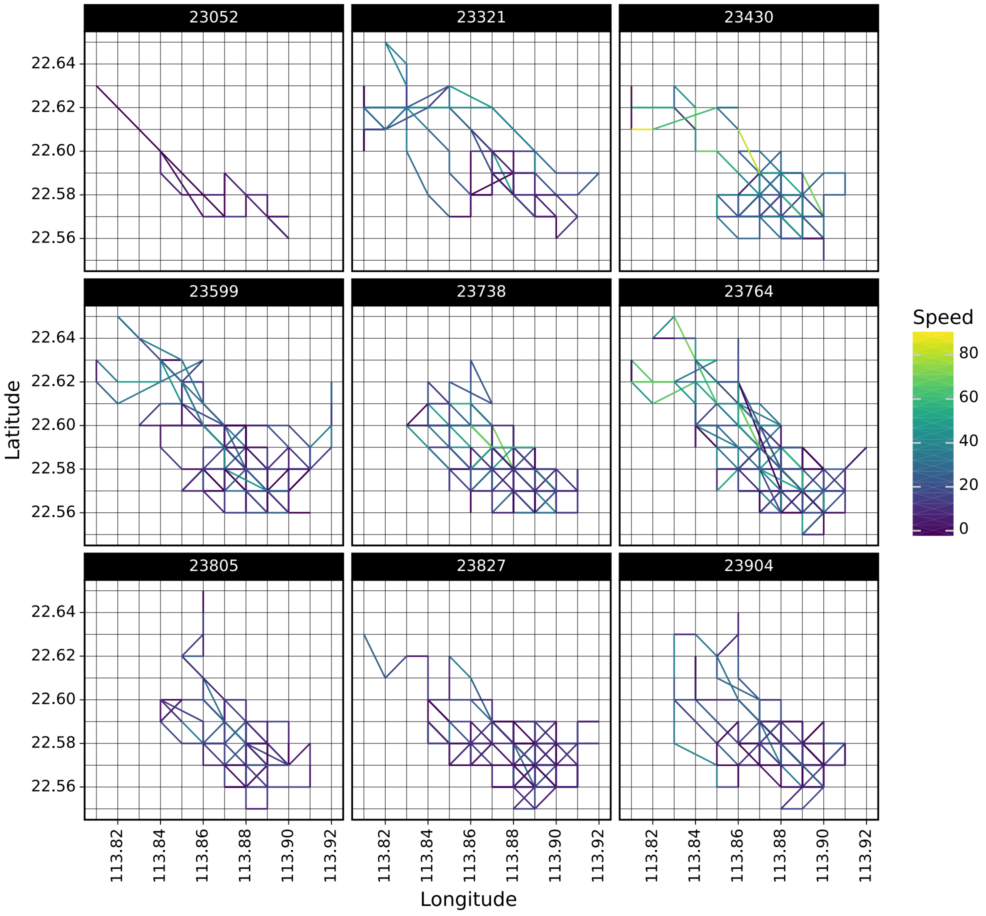

Out[30]: <ggplot: (145493012770)>

In [32]:

```
df_agg = mydf.groupby(by=['T', 'Longitude', 'Latitude']).count()
df_agg.reset_index(inplace=True)
# df_agg['Speed'].hist(bins=20, range=(0,20))

chart = (
    ggplot(df_agg)
    + geom_histogram(aes(x='Speed'), binwidth=1)
    + theme_linedraw()
    + xlab('Vehicle within Grid')
    + ylab('Time-space Grids')
    + theme(figure_size=(5, 2))
    + scale_x_continuous(breaks=range(0,20))
)

chart.save("histogram-ncar-73-cars")
chart
```

```
C:\Users\hshao33\AppData\Roaming\Python\Python38\site-packages\plotnine\ggplot.py:719: PlotnineWarning: Saving 5 x 2 in image.
C:\Users\hshao33\AppData\Roaming\Python\Python38\site-packages\plotnine\ggplot.py:722: PlotnineWarning: Filename: histogram-ncar-73
-cars
```

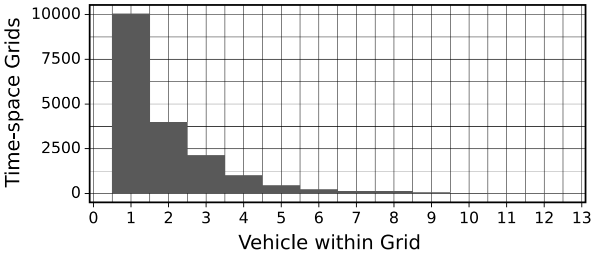

Out[32]: <ggplot: (145492492273)>

In [39]:
```
df_agg = df_agg.reset_index()

print('Number of Geographic Grids : ', df_agg.groupby(by=['Longitude', 'Latitude']).count().shape[0])
print('Number of Time Periods     : ', df_agg.groupby(by=['T']).count().shape[0])
```

```
Number of Geographic Grids :  164
Number of Time Periods     :  721
```

Optimization Models

In [1]:
```
import numpy as np
import cvxpy as cp
import pandas as pd

from plotnine import *

cp.installed_solvers()
```

Out[1]:
```
['CPLEX', 'ECOS', 'ECOS_BB', 'GUROBI', 'OSQP', 'SCIPY', 'SCS']
```

Read data frame and fill nan values.

In [12]:
```
mydf = pd.read_csv("gps-data-shenzhen-taxi-small-73.csv")

# fill nan values with previous values or future values or 0
df_temp = pd.DataFrame(np.array(
                            np.meshgrid(mydf['T'].unique(),
                                        mydf['CarID'].unique())
                          ).T.reshape(-1,2),
                          columns = ['T', 'CarID'])

mydf = pd.merge(df_temp, mydf, left_on=['T', 'CarID'], right_on=['T', 'CarID'], how='outer')
mydf.sort_values(by=['CarID', 'T', 'Longitude', 'Latitude'], inplace=True)

mydf['Speed'] = mydf.groupby(['CarID'])['Speed'].fillna(0)
mydf['Occupied'] = mydf.groupby(['CarID'])['Occupied'].fillna(0)
mydf['Longitude'] = mydf.groupby(['CarID'])['Longitude'].fillna(method="ffill").fillna(method="bfill")
mydf['Latitude'] = mydf.groupby(['CarID'])['Latitude'].fillna(method="ffill").fillna(method="bfill")

mydf
```

Out[12]:

	T	CarID	Longitude	Latitude	Occupied	Speed
**57**	0	22594	113.90	22.55	0.0	0.0
**130**	1	22594	113.90	22.55	0.0	0.0
**203**	2	22594	113.90	22.55	0.0	0.0
**276**	3	22594	113.90	22.55	0.0	0.0
**349**	4	22594	113.90	22.55	0.0	0.0
**...**	...	...	...	...	...	...
**52320**	716	35731	113.91	22.57	0.0	0.0
**52393**	717	35731	113.91	22.57	0.0	0.0
**52466**	718	35731	113.91	22.57	0.0	0.0
**52539**	719	35731	113.91	22.57	0.0	0.0
**52612**	720	35731	113.91	22.57	0.0	0.0

52633 rows × 6 columns

In [13]:
```
print('Total number of records. : ', mydf.shape[0])
print('Total number of vehicles : ', len(mydf['CarID'].unique()))
```

```
Total number of records. :  52633
Total number of vehicles :  73
```

Data Preparation

In [14]:
```
# velocity of cars over time
V = pd.pivot_table(mydf.drop(['Longitude', 'Latitude'], axis=1),
                   values='Speed',
                   index=['T'],
                   columns=['CarID'],
                   aggfunc=np.mean,
                   fill_value=0).values

print(np.sum(V) / 30 / 720)

V
```

```
11.35084027777778
```

Out[14]:
```
array([[ 0.  ,  0.  ,  0.  , ...,  0.  ,  0.  ,  0.  ],
       [ 0.  ,  0.  ,  0.  , ...,  0.  ,  0.  ,  0.  ],
       [ 0.  ,  0.  ,  0.  , ..., 10.33,  0.  ,  0.  ],
       ...,
       [ 0.  ,  0.  ,  0.  , ...,  0.  ,  0.  ,  0.  ],
       [ 0.  ,  0.  ,  0.  , ...,  0.  ,  0.  ,  0.  ],
       [ 0.  ,  0.  ,  0.  , ...,  0.  ,  0.  ,  0.  ]])
```

Indicators whether a car can get charged from charging stations / other cars

In [15]:
```
df_grids = mydf[['CarID', 'Longitude', 'Latitude']].drop_duplicates().copy()

I_stations = []
I_btwncars = []

for t in mydf['T'].unique():
  df_temp = mydf[mydf['T'] == t].copy()
  df_temp = pd.merge(df_temp, df_grids, left_on=['CarID', 'Longitude', 'Latitude'], right_on=['CarID', 'Longitude', 'Latitude'], how='
  df_temp = pd.pivot_table(df_temp,
                           values='Speed',
                           index=['CarID'],
                           columns=['Longitude', 'Latitude'],
                           aggfunc=np.max,
                           dropna = False,
                           fill_value=-1).values

  I_stations.append((df_temp == 0).astype(int))
  I_btwncars.append((df_temp >= 0).astype(int))
```

In [16]:
```
np.sum(I_btwncars[4], axis=0)
```

Out[16]:
```
array([0, 0, 0, 0, 0, 0, 0, 0, 0, 0, 1, 4, 0, 0, 0, 0, 0, 0, 0, 0, 0, 0,
       0, 0, 0, 0, 0, 2, 0, 0, 0, 0, 0, 0, 0, 0, 1, 0, 0, 0, 0, 0, 0, 0,
       0, 0, 0, 0, 0, 0, 3, 2, 0, 0, 0, 0, 0, 0, 0, 0, 0, 0, 0, 0, 0, 0,
       0, 0, 0, 0, 0, 0, 0, 0, 0, 0, 1, 0, 0, 1, 0, 1, 0, 1, 0, 0, 0, 1,
       0, 2, 1, 2, 1, 0, 0, 0, 0, 0, 0, 0, 0, 0, 1, 3, 8, 1, 0, 0, 0, 0,
       0, 0, 0, 0, 0, 1, 2, 4, 2, 5, 0, 0, 0, 0, 0, 0, 0, 0, 0, 1, 1, 1,
       2, 1, 0, 0, 0, 0, 0, 0, 0, 0, 1, 0, 2, 1, 8, 1, 0, 0, 0, 0, 0, 0,
       1, 1, 0, 1, 0, 0, 0, 0, 0, 0, 0, 0, 0])
```

Optimization - Min Battery by Min Charging

In [17]:
```
import datetime

# energy comsumption rate
# (or fraction of an hour, if battery level is measured
#  by distance it can travel without charging)
par_tau = 1 / 30
```

```
# ----------  ---------- #

# decision variable: battery levels
b = cp.Variable((V.shape[0]+1, V.shape[1]))
c = cp.Variable(I_stations[0].shape[1])

# decision variable: charging plan from stations / cars
u = []
v_in = []
v_out = []
for t in range(V.shape[0]):
  u.append(cp.Variable((I_stations[0].shape[0],1)))
  v_in.append(cp.Variable((I_btwncars[0].shape[0],1)))
  v_out.append(cp.Variable((I_btwncars[0].shape[0],1)))

# objective function:
objective = cp.max(b)



# ----------  ---------- #

# list for holding results
results = []



# battery charging rate form stations

for par_beta in np.array([2]):
  for par_theta in np.array([0, 0.5, 0.75]):
    print()
    print('Starting New Test: ', datetime.datetime.now())
    print('beta = ', par_beta, ' ; ', 'theta = ', par_theta)

    y = 11.4
    step = 0

    for k in range(50):
      y = y + step
      step = step + 0.1

      # add constraints
      constraints = [
        cp.sum(b[0,]) <= cp.sum(b[-1,]),
        cp.sum(c) <= y,
        c >= 0,
        b >= 160
      ]

      for t in range(V.shape[0]):
        constraints += [
          b[t+1,] == b[t,] - (par_tau * V[t,]) + u[t][:,0] + v_in[t][:,0] - v_out[t][:,0],
          cp.multiply(1 - np.sum(I_stations[t], axis=1), u[t][:,0]) == 0,
          cp.sum(cp.multiply(I_stations[t], cp.kron(np.ones((1, I_stations[0].shape[1])), u[t])), axis=0) <= c,
          cp.sum(cp.multiply(I_btwncars[t], cp.kron(np.ones((1, I_btwncars[0].shape[1])), v_in[t])), axis=0)
              <= par_theta * cp.sum(cp.multiply(I_btwncars[t], cp.kron(np.ones((1, I_btwncars[0].shape[1])), v_out[t])), axis=0),
          v_out[t] <= 2,
          v_in[t] <= 2,
          u[t] <= par_beta,
          v_out[t] >= 0,
          v_in[t] >= 0,
          u[t] >= 0
        ]

      # ---------- #

      # solve optimization problem 1
      problem = cp.Problem(cp.Minimize(objective), constraints)
      problem.solve(solver=cp.GUROBI, reoptimize=True)  # , reoptimize=True

      print('ending time of model 1: ', datetime.datetime.now())
      print('charging capacity     : ', y)
      print('objective value       : ', objective.value)


      if objective.value is not None:
        results.append(['With V-to-V Charging', par_beta, par_theta, y, objective.value])


      # # add additional constraints
      # for t in range(V.shape[0]):
      #   constraints += [
      #     v_out[t] == 0
      #   ]
```

```
    # # solve optimization problem 2
    # problem = cp.Problem(cp.Minimize(objective), constraints)
    # problem.solve(solver=cp.GUROBI, reoptimize=True)

    # print('ending time of model 2: ', datetime.datetime.now())
    # print('objective value       : ', objective.value)

    # if objective.value is not None:
    #   results.append(['Without V-V Charging', par_beta, par_theta, y, objective.value])



# ----------  ---------- #
```

```
4
ending time of model 1:  2022-03-11 11:50:29.711275
charging capacity     :  11.7
objective value       :  356.5506304688847
ending time of model 1:  2022-03-11 11:52:24.002865
charging capacity     :  12.0
objective value       :  349.9883410945504
ending time of model 1:  2022-03-11 11:54:23.739008
charging capacity     :  12.4
objective value       :  343.16697537830623
ending time of model 1:  2022-03-11 11:56:44.644483
charging capacity     :  12.9
objective value       :  336.3806395029043
ending time of model 1:  2022-03-11 11:58:54.059602
charging capacity     :  13.5
objective value       :  330.6626951150137
ending time of model 1:  2022-03-11 12:00:58.335346
charging capacity     :  14.2
objective value       :  324.02493753758563
ending time of model 1:  2022-03-11 12:03:13.674186
charging capacity     :  15.0
objective value       :  317.92811913618476
ending time of model 1:  2022-03-11 12:05:23.785245
charging capacity     :  15.9
objective value       :  311.69183090632197
ending time of model 1:  2022-03-11 12:07:36.689400
charging capacity     :  16.9
objective value       :  304.942507568096
ending time of model 1:  2022-03-11 12:09:53.588755
charging capacity     :  18.0
objective value       :  299.1447939338499
ending time of model 1:  2022-03-11 12:12:07.592694
charging capacity     :  19.2
objective value       :  294.2480815655944
ending time of model 1:  2022-03-11 12:14:24.041763
charging capacity     :  20.5
objective value       :  289.01538574693774
ending time of model 1:  2022-03-11 12:16:41.938974
charging capacity     :  21.9
objective value       :  283.4153710235841
ending time of model 1:  2022-03-11 12:19:01.648062
charging capacity     :  23.4
objective value       :  277.46135147549273
ending time of model 1:  2022-03-11 12:21:13.374210
charging capacity     :  25.0
objective value       :  272.56180434782607
ending time of model 1:  2022-03-11 12:23:23.272962
charging capacity     :  26.7
objective value       :  267.46743926309733
ending time of model 1:  2022-03-11 12:25:24.641189
charging capacity     :  28.5
objective value       :  262.171233201581
ending time of model 1:  2022-03-11 12:27:31.516070
charging capacity     :  30.400000000000002
objective value       :  256.60077865612647
ending time of model 1:  2022-03-11 12:29:36.481891
charging capacity     :  32.400000000000006
objective value       :  251.12152590342885
ending time of model 1:  2022-03-11 12:31:40.855120
charging capacity     :  34.50000000000001
objective value       :  246.71940725623583
ending time of model 1:  2022-03-11 12:33:46.635328
charging capacity     :  36.70000000000001
objective value       :  242.3105045045045
ending time of model 1:  2022-03-11 12:35:54.677304
charging capacity     :  39.000000000000014
objective value       :  238.4113333333334
ending time of model 1:  2022-03-11 12:38:02.093006
charging capacity     :  41.40000000000001
objective value       :  236.01133333333343
ending time of model 1:  2022-03-11 12:40:09.625630
charging capacity     :  43.90000000000001
objective value       :  233.5113333333334
ending time of model 1:  2022-03-11 12:42:17.256854
charging capacity     :  46.500000000000014
```

```
objective value       :  230.91133333333335
ending time of model 1:  2022-03-11 12:44:24.325061
charging capacity     :  49.20000000000002
objective value       :  228.2113333333334
ending time of model 1:  2022-03-11 12:46:33.009975
charging capacity     :  52.00000000000002
objective value       :  225.41133333333337
ending time of model 1:  2022-03-11 12:48:47.643901
charging capacity     :  54.90000000000002
objective value       :  223.6050000000001
ending time of model 1:  2022-03-11 12:51:01.064523
charging capacity     :  57.90000000000002
objective value       :  223.6050000000001
ending time of model 1:  2022-03-11 12:53:11.260048
charging capacity     :  61.00000000000002
objective value       :  223.6050000000001
ending time of model 1:  2022-03-11 12:55:15.076915
charging capacity     :  64.20000000000002
objective value       :  223.6050000000001
ending time of model 1:  2022-03-11 12:57:26.859256
charging capacity     :  67.50000000000001
objective value       :  223.6050000000001
ending time of model 1:  2022-03-11 12:59:34.796576
charging capacity     :  70.90000000000002
objective value       :  223.6050000000001
ending time of model 1:  2022-03-11 13:01:36.653832
charging capacity     :  74.40000000000002
objective value       :  223.6050000000001
ending time of model 1:  2022-03-11 13:03:40.176095
charging capacity     :  78.00000000000003
objective value       :  223.6050000000001
ending time of model 1:  2022-03-11 13:05:40.763300
charging capacity     :  81.70000000000003
objective value       :  223.6050000000001
ending time of model 1:  2022-03-11 13:07:39.508013
charging capacity     :  85.50000000000003
objective value       :  223.6050000000001
ending time of model 1:  2022-03-11 13:09:38.604498
charging capacity     :  89.40000000000003
objective value       :  223.6050000000001
ending time of model 1:  2022-03-11 13:11:36.601997
charging capacity     :  93.40000000000003
objective value       :  223.6050000000001
ending time of model 1:  2022-03-11 13:13:37.350474
charging capacity     :  97.50000000000003
objective value       :  223.6050000000001
ending time of model 1:  2022-03-11 13:15:40.721943
charging capacity     :  101.70000000000003
objective value       :  223.6050000000001
ending time of model 1:  2022-03-11 13:17:45.865405
charging capacity     :  106.00000000000003
objective value       :  223.6050000000001
ending time of model 1:  2022-03-11 13:19:49.928872
charging capacity     :  110.40000000000003
objective value       :  223.6050000000001
ending time of model 1:  2022-03-11 13:21:49.308360
charging capacity     :  114.90000000000003
objective value       :  223.6050000000001
ending time of model 1:  2022-03-11 13:23:53.226827
charging capacity     :  119.50000000000003
objective value       :  223.6050000000001
ending time of model 1:  2022-03-11 13:25:57.034300
charging capacity     :  124.20000000000003
objective value       :  223.6050000000001
ending time of model 1:  2022-03-11 13:27:57.618780
charging capacity     :  129.00000000000003
objective value       :  223.6050000000001
ending time of model 1:  2022-03-11 13:29:55.964270
charging capacity     :  133.90000000000003
objective value       :  223.6050000000001

Starting New Test:  2022-03-11 13:29:55.965265
beta =  2  ;  theta =  0.5
ending time of model 1:  2022-03-11 13:32:28.482624
charging capacity     :  11.4
objective value       :  406.27239184101836
ending time of model 1:  2022-03-11 13:35:14.672945
charging capacity     :  11.5
objective value       :  341.6187876081782
ending time of model 1:  2022-03-11 13:37:44.198313
charging capacity     :  11.7
objective value       :  288.57028715243456
ending time of model 1:  2022-03-11 13:40:26.745615
charging capacity     :  12.0
objective value       :  237.2427049961224
ending time of model 1:  2022-03-11 13:43:15.891887
charging capacity     :  12.4
objective value       :  193.39818077914356
ending time of model 1:  2022-03-11 13:46:06.613153
charging capacity     :  12.9
objective value       :  158.2003136479125
```

```
ending time of model 1:  2022-03-11 13:48:42.510509
charging capacity     :  13.5
objective value       :  126.17613925393081
ending time of model 1:  2022-03-11 13:51:38.833785
charging capacity     :  14.2
objective value       :  101.01635511384528
ending time of model 1:  2022-03-11 13:54:28.383059
charging capacity     :  15.0
objective value       :  79.99926079361454
ending time of model 1:  2022-03-11 13:57:37.608239
charging capacity     :  15.9
objective value       :  62.334012020588744
ending time of model 1:  2022-03-11 14:00:26.321516
charging capacity     :  16.9
objective value       :  50.23295186679951
ending time of model 1:  2022-03-11 14:03:31.625746
charging capacity     :  18.0
objective value       :  42.142250655137794
ending time of model 1:  2022-03-11 14:06:39.506970
charging capacity     :  19.2
objective value       :  36.13885293857446
ending time of model 1:  2022-03-11 14:09:29.075239
charging capacity     :  20.5
objective value       :  31.705629988402936
ending time of model 1:  2022-03-11 14:12:31.511462
charging capacity     :  21.9
objective value       :  29.423067654551453
ending time of model 1:  2022-03-11 14:15:49.102609
charging capacity     :  23.4
objective value       :  28.411067591063627
ending time of model 1:  2022-03-11 14:18:54.204843
charging capacity     :  25.0
objective value       :  27.499051674643443
ending time of model 1:  2022-03-11 14:21:40.295158
charging capacity     :  26.7
objective value       :  26.572707564735314
ending time of model 1:  2022-03-11 14:24:06.587529
charging capacity     :  28.5
objective value       :  25.671333333333337
ending time of model 1:  2022-03-11 14:26:28.982919
charging capacity     :  30.400000000000002
objective value       :  25.671333333333337
ending time of model 1:  2022-03-11 14:28:49.582312
charging capacity     :  32.400000000000006
objective value       :  25.671333333333333
ending time of model 1:  2022-03-11 14:31:08.223716
charging capacity     :  34.50000000000001
objective value       :  25.671333333333333
ending time of model 1:  2022-03-11 14:33:28.044118
charging capacity     :  36.70000000000001
objective value       :  25.671333333333333
ending time of model 1:  2022-03-11 14:35:41.947540
charging capacity     :  39.000000000000014
objective value       :  25.671333333333333
ending time of model 1:  2022-03-11 14:37:59.550968
charging capacity     :  41.40000000000001
objective value       :  25.671333333333333
ending time of model 1:  2022-03-11 14:40:18.484398
charging capacity     :  43.90000000000001
objective value       :  25.671333333333333
ending time of model 1:  2022-03-11 14:42:34.534825
charging capacity     :  46.500000000000014
objective value       :  25.671333333333333
ending time of model 1:  2022-03-11 14:44:53.935223
charging capacity     :  49.20000000000002
objective value       :  25.671333333333333
ending time of model 1:  2022-03-11 14:47:12.508634
charging capacity     :  52.00000000000002
objective value       :  25.671333333333333
ending time of model 1:  2022-03-11 14:49:32.280029
charging capacity     :  54.90000000000002
objective value       :  25.671333333333333
ending time of model 1:  2022-03-11 14:51:47.035455
charging capacity     :  57.90000000000002
objective value       :  25.671333333333333
ending time of model 1:  2022-03-11 14:54:05.247885
charging capacity     :  61.00000000000002
objective value       :  25.671333333333333
ending time of model 1:  2022-03-11 14:56:25.729311
charging capacity     :  64.20000000000002
objective value       :  25.671333333333333
ending time of model 1:  2022-03-11 14:58:38.300744
charging capacity     :  67.50000000000001
objective value       :  25.671333333333333
ending time of model 1:  2022-03-11 15:00:47.088187
charging capacity     :  70.90000000000002
objective value       :  25.671333333333333
ending time of model 1:  2022-03-11 15:02:57.225628
charging capacity     :  74.40000000000002
objective value       :  25.671333333333333
ending time of model 1:  2022-03-11 15:05:08.256068
```

```
charging capacity     :  78.00000000000003
objective value       :  25.671333333333333
ending time of model 1:  2022-03-11 15:07:14.660532
charging capacity     :  81.70000000000003
objective value       :  25.671333333333333
ending time of model 1:  2022-03-11 15:09:20.277018
charging capacity     :  85.50000000000003
objective value       :  25.671333333333333
ending time of model 1:  2022-03-11 15:11:28.446490
charging capacity     :  89.40000000000003
objective value       :  25.671333333333333
ending time of model 1:  2022-03-11 15:13:39.962927
charging capacity     :  93.40000000000003
objective value       :  25.671333333333333
ending time of model 1:  2022-03-11 15:15:47.885369
charging capacity     :  97.50000000000003
objective value       :  25.671333333333333
ending time of model 1:  2022-03-11 15:17:57.554814
charging capacity     :  101.70000000000003
objective value       :  25.671333333333333
ending time of model 1:  2022-03-11 15:20:06.496262
charging capacity     :  106.00000000000003
objective value       :  25.671333333333333
ending time of model 1:  2022-03-11 15:22:11.274736
charging capacity     :  110.40000000000003
objective value       :  25.671333333333333
ending time of model 1:  2022-03-11 15:24:14.050780
charging capacity     :  114.90000000000003
objective value       :  25.671333333333333
ending time of model 1:  2022-03-11 15:26:25.953529
charging capacity     :  119.50000000000003
objective value       :  25.671333333333333
ending time of model 1:  2022-03-11 15:28:37.373301
charging capacity     :  124.20000000000003
objective value       :  25.671333333333333
ending time of model 1:  2022-03-11 15:30:44.799139
charging capacity     :  129.00000000000003
objective value       :  25.671333333333333
ending time of model 1:  2022-03-11 15:32:58.973925
charging capacity     :  133.90000000000003
objective value       :  25.671333333333333

Starting New Test:  2022-03-11 15:32:58.974920
beta =  2  ;  theta =  0.75
ending time of model 1:  2022-03-11 15:35:42.084487
charging capacity     :  11.4
objective value       :  339.6731784973918
ending time of model 1:  2022-03-11 15:38:18.284124
charging capacity     :  11.5
objective value       :  269.98487397026844
ending time of model 1:  2022-03-11 15:40:51.594834
charging capacity     :  11.7
objective value       :  197.47313969582652
ending time of model 1:  2022-03-11 15:43:55.475314
charging capacity     :  12.0
objective value       :  140.88502155124095
ending time of model 1:  2022-03-11 15:46:41.780960
charging capacity     :  12.4
objective value       :  99.73869659977427
ending time of model 1:  2022-03-11 15:49:33.137593
charging capacity     :  12.9
objective value       :  68.50304958414075
ending time of model 1:  2022-03-11 15:52:33.622169
charging capacity     :  13.5
objective value       :  46.54291480326072
ending time of model 1:  2022-03-11 15:55:25.211861
charging capacity     :  14.2
objective value       :  34.432583132401255
ending time of model 1:  2022-03-11 15:58:26.872489
charging capacity     :  15.0
objective value       :  27.372600686890966
ending time of model 1:  2022-03-11 16:01:42.514062
charging capacity     :  15.9
objective value       :  26.024260312107867
ending time of model 1:  2022-03-11 16:04:46.777738
charging capacity     :  16.9
objective value       :  25.204832354433965
ending time of model 1:  2022-03-11 16:07:49.066444
charging capacity     :  18.0
objective value       :  24.336710955708508
ending time of model 1:  2022-03-11 16:10:41.952250
charging capacity     :  19.2
objective value       :  23.417060864228432
ending time of model 1:  2022-03-11 16:13:05.929259
charging capacity     :  20.5
objective value       :  23.171333333333333
ending time of model 1:  2022-03-11 16:15:30.745290
charging capacity     :  21.9
objective value       :  23.171333333333333
ending time of model 1:  2022-03-11 16:17:52.784337
charging capacity     :  23.4
```

```
objective value       :  23.171333333333333
ending time of model 1:  2022-03-11 16:20:14.472419
charging capacity     :  25.0
objective value       :  23.171333333333333
ending time of model 1:  2022-03-11 16:22:41.196470
charging capacity     :  26.7
objective value       :  23.171333333333333
ending time of model 1:  2022-03-11 16:25:02.257562
charging capacity     :  28.5
objective value       :  23.171333333333333
ending time of model 1:  2022-03-11 16:27:23.057658
charging capacity     :  30.400000000000002
objective value       :  23.171333333333333
ending time of model 1:  2022-03-11 16:29:43.306784
charging capacity     :  32.400000000000006
objective value       :  23.171333333333333
ending time of model 1:  2022-03-11 16:32:01.593926
charging capacity     :  34.50000000000001
objective value       :  23.171333333333333
ending time of model 1:  2022-03-11 16:34:18.589088
charging capacity     :  36.70000000000001
objective value       :  23.171333333333333
ending time of model 1:  2022-03-11 16:36:40.351224
charging capacity     :  39.000000000000014
objective value       :  23.171333333333333
ending time of model 1:  2022-03-11 16:38:56.294410
charging capacity     :  41.40000000000001
objective value       :  23.171333333333333
ending time of model 1:  2022-03-11 16:41:13.453596
charging capacity     :  43.90000000000001
objective value       :  23.171333333333333
ending time of model 1:  2022-03-11 16:43:27.757809
charging capacity     :  46.500000000000014
objective value       :  23.171333333333333
ending time of model 1:  2022-03-11 16:45:40.820039
charging capacity     :  49.20000000000002
objective value       :  23.171333333333333
ending time of model 1:  2022-03-11 16:47:58.643238
charging capacity     :  52.00000000000002
objective value       :  23.171333333333333
ending time of model 1:  2022-03-11 16:50:14.938462
charging capacity     :  54.90000000000002
objective value       :  23.171333333333333
ending time of model 1:  2022-03-11 16:52:32.501675
charging capacity     :  57.90000000000002
objective value       :  23.171333333333333
ending time of model 1:  2022-03-11 16:54:50.524901
charging capacity     :  61.00000000000002
objective value       :  23.171333333333333
ending time of model 1:  2022-03-11 16:57:10.165119
charging capacity     :  64.20000000000002
objective value       :  23.171333333333333
ending time of model 1:  2022-03-11 16:59:32.249335
charging capacity     :  67.50000000000001
objective value       :  23.171333333333333
ending time of model 1:  2022-03-11 17:01:51.247575
charging capacity     :  70.90000000000002
objective value       :  23.171333333333333
ending time of model 1:  2022-03-11 17:04:03.732848
charging capacity     :  74.40000000000002
objective value       :  23.171333333333333
ending time of model 1:  2022-03-11 17:06:15.055138
charging capacity     :  78.00000000000003
objective value       :  23.171333333333333
ending time of model 1:  2022-03-11 17:08:29.334418
charging capacity     :  81.70000000000003
objective value       :  23.171333333333333
ending time of model 1:  2022-03-11 17:10:42.676714
charging capacity     :  85.50000000000003
objective value       :  23.171333333333333
ending time of model 1:  2022-03-11 17:12:52.477024
charging capacity     :  89.40000000000003
objective value       :  23.171333333333333
ending time of model 1:  2022-03-11 17:15:01.680351
charging capacity     :  93.40000000000003
objective value       :  23.171333333333333
ending time of model 1:  2022-03-11 17:17:08.668696
charging capacity     :  97.50000000000003
objective value       :  23.171333333333333
ending time of model 1:  2022-03-11 17:19:16.793025
charging capacity     :  101.70000000000003
objective value       :  23.171333333333333
ending time of model 1:  2022-03-11 17:21:26.092352
charging capacity     :  106.00000000000003
objective value       :  23.171333333333333
ending time of model 1:  2022-03-11 17:23:30.334709
charging capacity     :  110.40000000000003
objective value       :  23.171333333333333
ending time of model 1:  2022-03-11 17:25:34.762060
charging capacity     :  114.90000000000003
objective value       :  23.171333333333333
```

```
ending time of model 1:  2022-03-11 17:27:38.439420
charging capacity     :  119.50000000000003
objective value       :  23.171333333333333
ending time of model 1:  2022-03-11 17:29:44.977774
charging capacity     :  124.20000000000003
objective value       :  23.171333333333333
ending time of model 1:  2022-03-11 17:31:50.438135
charging capacity     :  129.00000000000003
objective value       :  23.171333333333333
ending time of model 1:  2022-03-11 17:33:56.174491
charging capacity     :  133.90000000000003
objective value       :  23.171333333333333
```

In [18]:

```
df_plot = pd.DataFrame(results, columns=['Case', 'Beta', 'Theta', 'Total Charging Capacity', 'Battery Capacity'])
df_plot['Total Charging Capacity'] = df_plot['Total Charging Capacity'].astype('float')
df_plot['Battery Capacity'] = df_plot['Battery Capacity'].astype('float')
df_plot['Theta'] = df_plot['Theta'].astype('float')
df_plot['Beta'] = df_plot['Beta'].astype('float')
df_plot['Case'] = df_plot['Case'].astype('string')

df_plot.to_csv("result-73cars-charging-to-battery.csv", index = False)

df_plot
```

Out[18]:

	Case	Beta	Theta	Total Charging Capacity	Battery Capacity
**0**	With V-to-V Charging	2.0	0.00	11.4	406.272392
**1**	With V-to-V Charging	2.0	0.00	11.5	361.010065
**2**	With V-to-V Charging	2.0	0.00	11.7	356.550630
**3**	With V-to-V Charging	2.0	0.00	12.0	349.988341
**4**	With V-to-V Charging	2.0	0.00	12.4	343.166975
**...**	...	...	...	...	...
**145**	With V-to-V Charging	2.0	0.75	114.9	23.171333
**146**	With V-to-V Charging	2.0	0.75	119.5	23.171333
**147**	With V-to-V Charging	2.0	0.75	124.2	23.171333
**148**	With V-to-V Charging	2.0	0.75	129.0	23.171333
**149**	With V-to-V Charging	2.0	0.75	133.9	23.171333

150 rows × 5 columns

In [42]:

```
from plotnine import *
from mizani.transforms  import log_trans

df_plot = pd.read_csv("result-73cars-charging-to-battery.csv")

df_plot['Total Charging Capacity'] = df_plot['Total Charging Capacity'] / 2
df_plot = df_plot[df_plot['Total Charging Capacity'] <= 42]
df_plot['Battery Capacity'] = df_plot['Battery Capacity'] + 160


df_plot = df_plot.assign(
    Scenario = lambda dataframe: dataframe['Theta'].map(lambda theta: 'No V2V Charging / V2V Charging with Efficiency 0.0%' if theta =
)

chart = (
    ggplot(df_plot[df_plot['Beta']==2.0])
    + geom_point(aes(x='Total Charging Capacity', y='Battery Capacity', color='Scenario'), size=0.5)
    + geom_line(aes(x='Total Charging Capacity', y='Battery Capacity', color='Scenario'))
    + geom_hline(yintercept = 400, linetype="dashed")
    + geom_vline(xintercept = 6, linetype="dashed")
    + geom_vline(xintercept = 20, linetype="dashed")
    + theme_linedraw()
    + xlab('Total Number of Charging Piles')
    + ylab('Battery Size / Range (in kilometers)')
    + theme(figure_size=(6, 3), legend_position=(0.5,1.2))
    + theme(axis_text_x = element_text(angle=90))
    # + scale_x_continuous(breaks=np.arange(5,8.2,0.2) / 10)
    + scale_x_continuous(trans = log_trans(base=10), breaks=np.array([6,10,20,30,40,50,60]))
    + scale_y_continuous(trans = log_trans(base=10), breaks=np.arange(0,700,100))
    # + scale_y_log10()
    # + scale_x_log10()
)

chart.save("result-new-normal2.png")

chart
```

```
C:\Users\hshao33\AppData\Roaming\Python\Python38\site-packages\plotnine\ggplot.py:719: PlotnineWarning: Saving 6 x 3 in image.
C:\Users\hshao33\AppData\Roaming\Python\Python38\site-packages\plotnine\ggplot.py:722: PlotnineWarning: Filename: result-new-normal
2.png
C:\Users\hshao33\AppData\Roaming\Python\Python38\site-packages\plotnine\scales\scale.py:697: RuntimeWarning: divide by zero encount
ered in log10
C:\Users\hshao33\AppData\Roaming\Python\Python38\site-packages\plotnine\scales\scale.py:697: RuntimeWarning: divide by zero encount
ered in log10
```

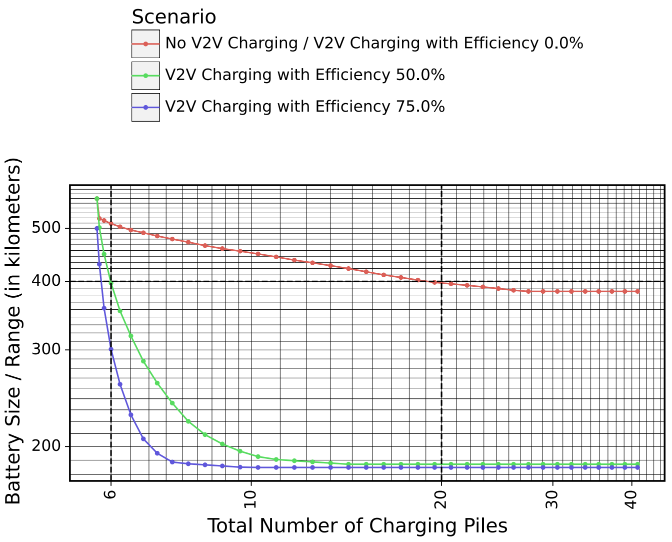

Out[42]: `<ggplot: (139843880470)>`

Optimization - Min Charging by Min Battery

In [ ]:

```
import datetime

# energy comsumption rate
# (or fraction of an hour, if battery level is measured
#  by distance it can travel without charging)
par_tau = 1 / 30



# ----------  ---------- #

# decision variable: battery levels
b = cp.Variable((V.shape[0]+1, V.shape[1]))
c = cp.Variable(I_stations[0].shape[1])

# decision variable: charging plan from stations / cars
u = []
v_in = []
v_out = []
for t in range(V.shape[0]):
  u.append(cp.Variable((I_stations[0].shape[0],1)))
  v_in.append(cp.Variable((I_btwncars[0].shape[0],1)))
  v_out.append(cp.Variable((I_btwncars[0].shape[0],1)))

# objective function:
objective = cp.sum(c)



# ----------  ---------- #

# list for holding results
results = []



# battery charging rate form stations

for par_beta in np.array([2, 20]):
  for par_theta in np.array([0, 0.25, 0.50, 0.75]):
```

```
    print()
    print('Starting New Test: ', datetime.datetime.now())
    print('beta = ', par_beta, ' ; ', 'theta = ', par_theta)
    print()
    for z in np.arange(100, 550, 50):

      # add constraints
      constraints = [
        cp.sum(b[0,]) <= cp.sum(b[-1,]),
        b <= z,
        b >= 160
      ]

      for t in range(V.shape[0]):
        constraints += [
          b[t+1,] == b[t,] - (par_tau * V[t,]) + u[t][:,0] + v_in[t][:,0] - v_out[t][:,0],
          cp.multiply(1 - np.sum(I_stations[t], axis=1), u[t][:,0]) == 0,
          cp.sum(cp.multiply(I_stations[t], cp.kron(np.ones((1, I_stations[0].shape[1])), u[t])), axis=0) <= c,
          cp.sum(cp.multiply(I_btwncars[t], cp.kron(np.ones((1, I_btwncars[0].shape[1])), v_in[t])), axis=0)
              <= par_theta * cp.sum(cp.multiply(I_btwncars[t], cp.kron(np.ones((1, I_btwncars[0].shape[1])), v_out[t])), axis=0),
          v_out[t] <= 2,
          v_in[t] <= 2,
          u[t] <= par_beta,
          v_out[t] >= 0,
          v_in[t] >= 0,
          u[t] >= 0
        ]

      # ---------- #

      # solve optimization problem 1
      problem = cp.Problem(cp.Minimize(objective), constraints)
      problem.solve(solver=cp.GUROBI, reoptimize=True)

      print('ending time of model 1: ', datetime.datetime.now())
      print('objective value       : ', objective.value)

      if objective.value is not None:
        results.append(['With V-to-V Charging', par_beta, par_theta, z, objective.value])


      # # add additional constraints
      # for t in range(V.shape[0]):
      #   constraints += [
      #     v_out[t] == 0
      #   ]

      # # solve optimization problem 2
      # problem = cp.Problem(cp.Minimize(objective), constraints)
      # prbblem.solve(solver=cp.GUROBI, reoptimize=True)

      # print('ending time of model 2: ', datetime.datetime.now())
      # print('objective value       : ', objective.value)

      # if objective.value is not None:
      #   results.append(['Without V-V Charging', par_beta, par_theta, z, objective.value])



# ----------  ---------- #
```

In [18]:

```
df_plot = pd.DataFrame(results, columns=['Case', 'Beta', 'Theta', 'Battery Capacity', 'Total Charging Capacity'])
df_plot['Total Charging Capacity'] = df_plot['Total Charging Capacity'].astype('float')
df_plot['Battery Capacity'] = df_plot['Battery Capacity'].astype('float')
df_plot['Theta'] = df_plot['Theta'].astype('float')
df_plot['Beta'] = df_plot['Beta'].astype('float')
df_plot['Case'] = df_plot['Case'].astype('string')

df_plot.to_csv("result-73cars-battery-to-charging.csv", index = False)

df_plot
```

Out[18]:

	Case	Beta	Theta	Battery Capacity	Total Charging Capacity
0	With V-to-V Charging	2.0	0.00	250.0	25.002076
1	With V-to-V Charging	2.0	0.00	300.0	14.513806
2	With V-to-V Charging	2.0	0.00	350.0	11.527263
3	With V-to-V Charging	2.0	0.00	400.0	11.287505
4	With V-to-V Charging	2.0	0.00	450.0	11.241537
...	...	...	...	...	...
64	With V-to-V Charging	20.0	0.75	300.0	11.284093
65	With V-to-V Charging	20.0	0.75	350.0	11.245436
66	With V-to-V Charging	20.0	0.75	400.0	11.233934
67	With V-to-V Charging	20.0	0.75	450.0	11.233934
68	With V-to-V Charging	20.0	0.75	500.0	11.233934

69 rows × 5 columns

In [97]:

```
df_plot = pd.read_csv("result-73cars-battery-to-charging.csv")
```

In [98]:

```
from plotnine import *

df_plot['Theta'] = df_plot['Theta'].astype('string')


chart = (
    ggplot(df_plot)
    + geom_point(aes(x='Battery Capacity', y='Total Charging Capacity', color = 'Theta')) # , color='Case'
    + geom_line(aes(x='Battery Capacity', y='Total Charging Capacity', color = 'Theta')) # , color='Case'
    + facet_grid('Beta ~ .')
    + theme_linedraw()
    + xlab('Battery Capacity (kilometers)')
    + ylab('Total Charging Capacity (kilometers per 2 minutes)')
    + theme(figure_size=(6, 4), legend_position='top')
    + xlim(100,500) + ylim(0,50)
    + theme(axis_text_x = element_text(angle=90))
)

chart.save("result-73cars-battery-to-charging.png")
chart
```

```
C:\Users\hshao33\AppData\Roaming\Python\Python38\site-packages\plotnine\ggplot.py:719: PlotnineWarning: Saving 6 x 4 in image.
C:\Users\hshao33\AppData\Roaming\Python\Python38\site-packages\plotnine\ggplot.py:722: PlotnineWarning: Filename: result-47cars-battery-to-charging.png
```

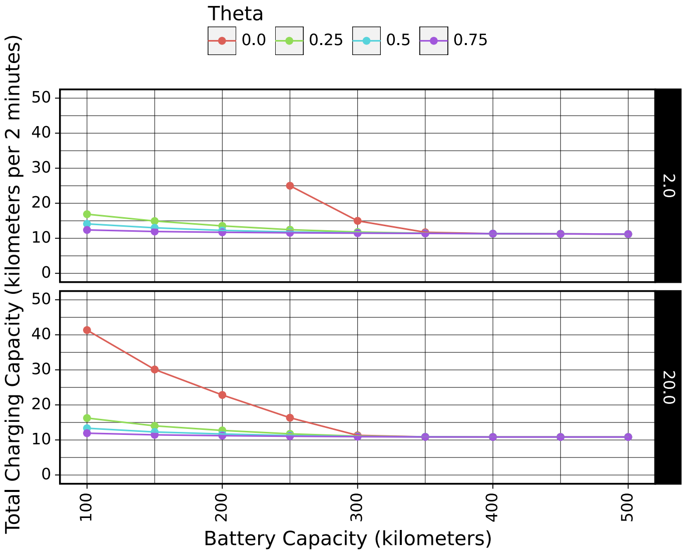

Out[98]: <ggplot: (94090487661)>

A Minimal Example

In [1]:
```
import numpy as np
import cvxpy as cp
import pandas as pd

from plotnine import *
```

Consider three locations: A, B, C, and three cars: 1, 2, 3. The cars either drive at a speed of 50 miles / hour, or stay in a parking lot with zero speed. The distance between A and B is 100 miles. The distance between B and C is 200 miles.

Car 1 starts from A, drives from A to B, then from B to C, stays at C for one hour, then drives from C to B, and stays at B for one hour.

Car 2 starts from A, drives from A to B, stays there afterwards.

Car 3 starts from B, and then joins Car 1 for the same travel plan afterwards.

Let 1 hour be the length of time periods. We consider a planning horizon of 12 hours.

In [ ]:

```
# energy comsumption rate
# (or fraction of an hour, if battery level is measured
#  by distance it can travel without charging)
par_tau = 1
# battery charging rate form stations
par_beta = 12
# charging efficiency between cars
par_theta = 0.5

# velocity of cars over time
V = np.array([[50, 50, 50, 50, 50, 50,  0, 50, 50, 50, 50,  0],
              [50, 50,  0,  0,  0,  0,  0,  0,  0,  0,  0,  0],
              [ 0,  0, 50, 50, 50, 50,  0, 50, 50, 50, 50,  0]])
V = np.transpose(V)


# indicator (if a car can get charged from a station)
I_stations = []
# indicator (if a car can get charged from another cars)
I_btwncars = []

for t in range(12):
  if t+1 in [1, 2]:
    # the columns corresponds to B, C, respectively
    I_stations.append(np.array([[0, 0],    # car 1
                                [0, 0],    # car 2
                                [1, 0]]))  # car 3
    # the columns corresponds to 1, 2, 3, respectively
    I_btwncars.append(np.array([[0, 1, 0],    # car 1
                                [1, 0, 0],    # car 2
                                [0, 0, 0]]))  # car 3
  elif t+1 in [7, 12]:
    # the columns corresponds to B, C, respectively
    I_stations.append(np.array([[0, 1],    # car 1
                                [1, 0],    # car 2
                                [0, 1]]))  # car 3
    # the columns corresponds to 1, 2, 3, respectively
    I_btwncars.append(np.array([[0, 0, 0],    # car 1
                                [0, 0, 0],    # car 2
                                [0, 0, 0]]))  # car 3
  else:
    # the columns corresponds to B, C, respectively
    I_stations.append(np.array([[0, 0],    # car 1
                                [1, 0],    # car 2
                                [0, 0]]))  # car 3
    # the columns corresponds to 1, 2, 3, respectively
    I_btwncars.append(np.array([[0, 0, 1],    # car 1
                                [0, 0, 0],    # car 2
                                [1, 0, 0]]))  # car 3

I_stations = np.asarray(I_stations)
I_btwncars = np.asarray(I_btwncars)
```

In [ ]:

```
# list for holding results
results = []


# ----------  ---------- #

# decision variable: battery levels
b = cp.Variable((V.shape[0]+1, V.shape[1]))
# decision variable: charging station capacities
c = cp.Variable(2)
# decision variable: charging plan from stations / cars
u = []
v = []

for t in range(12):
  u.append(cp.Variable(I_stations[0].shape))
  v.append(cp.Variable(I_btwncars[0].shape))

# objective function:
objective = cp.sum(c)
# objective = cp.max(b)


# ----------  ---------- #

for z in np.arange(0, 1000, 50):
  # constraints
  constraints = [
    cp.max(b) <= z,
    cp.sum(b[0,]) == cp.sum(b[-1,]),
    # cp.sum(c) <= y,
    b >= 0,
    c >= 0
  ]

  # ---------- #

  for t in range(12):
    constraints += [
      b[t+1,] == b[t,] - (par_tau * V[t,])
        - cp.sum(cp.multiply(I_btwncars[t], v[t]), axis=0)
        + cp.sum(cp.multiply(I_btwncars[t], v[t]), axis=1) * par_theta
        + cp.sum(cp.multiply(I_stations[t], u[t]), axis=1),
      cp.sum(cp.multiply(I_stations[t], u[t]), axis=0) <= c,
      u[t] >= 0,
      v[t] >= 0
    ]

  problem = cp.Problem(cp.Minimize(objective), constraints)
  problem.solve(solver='ECOS')
  if objective.value is not None:
    results.append(['With V-to-V Charging', z, objective.value])

  for t in range(12):
    constraints += [
      v[t] == 0
    ]

  problem = cp.Problem(cp.Minimize(objective), constraints)
  problem.solve(solver='ECOS')
  if objective.value is not None:
    results.append(['Without V-V Charging', z, objective.value])



np.set_printoptions(suppress=True)
np.set_printoptions(precision=0)
results
```

```
Out[ ]: [['With V-to-V Charging', 250, 400.0000002260023],
 ['With V-to-V Charging', 300, 350.0000000268239],
 ['Without V-V Charging', 300, 350.00000000012426],
 ['With V-to-V Charging', 350, 325.00000023028133],
 ['Without V-V Charging', 350, 325.0000000005469],
 ['With V-to-V Charging', 400, 300.00000026324426],
 ['Without V-V Charging', 400, 300.00000000101284],
 ['With V-to-V Charging', 450, 275.0000002737963],
 ['Without V-V Charging', 450, 275.0000000004525],
 ['With V-to-V Charging', 500, 250.00000025083065],
 ['Without V-V Charging', 500, 250.0000000084257],
 ['With V-to-V Charging', 550, 225.0000003906047],
 ['Without V-V Charging', 550, 225.00000000043306],
 ['With V-to-V Charging', 600, 200.00000000572768],
 ['Without V-V Charging', 600, 200.0000000011638],
 ['With V-to-V Charging', 650, 175.00000000916114],
 ['Without V-V Charging', 650, 175.0000000018513],
 ['With V-to-V Charging', 700, 150.0000000147832],
 ['Without V-V Charging', 700, 150.000000001429],
 ['With V-to-V Charging', 750, 125.00000004178848],
 ['Without V-V Charging', 750, 125.00000001716694],
 ['With V-to-V Charging', 800, 100.00000034204301],
 ['Without V-V Charging', 800, 100.00000000043082],
 ['With V-to-V Charging', 850, 83.3333337400008],
 ['Without V-V Charging', 850, 83.33333333416007],
 ['With V-to-V Charging', 900, 83.33333334313595],
 ['Without V-V Charging', 900, 83.33333336830549],
 ['With V-to-V Charging', 950, 83.33333400545335],
 ['Without V-V Charging', 950, 83.3333333631055]]
```

In [ ]:

```
# list for holding results
results = []


# ----------  ---------- #

# decision variable: battery levels
b = cp.Variable((V.shape[0]+1, V.shape[1]))
# decision variable: charging station capacities
c = cp.Variable(2)
# decision variable: charging plan from stations / cars
u = []
v = []

for t in range(12):
  u.append(cp.Variable(I_stations[0].shape))
  v.append(cp.Variable(I_btwncars[0].shape))

# objective function:
# objective = cp.sum(c)
objective = cp.max(b)


for y in np.arange(0, 1000, 50):
  # constraints
  constraints = [
    # cp.max(b) <= z,
    cp.sum(b[0,]) == cp.sum(b[-1,]),
    cp.sum(c) <= y,
    b >= 0,
    c >= 0
  ]

  # ---------- #

  for t in range(12):
    constraints += [
      b[t+1,] == b[t,] - (par_tau * V[t,])
        - cp.sum(cp.multiply(I_btwncars[t], v[t]), axis=0)
        + cp.sum(cp.multiply(I_btwncars[t], v[t]), axis=1) * par_theta
        + cp.sum(cp.multiply(I_stations[t], u[t]), axis=1),
      cp.sum(cp.multiply(I_stations[t], u[t]), axis=0) <= c,
      u[t] >= 0,
      v[t] >= 0
    ]

  problem = cp.Problem(cp.Minimize(objective), constraints)
  problem.solve(solver='ECOS')
  if objective.value is not None:
    results.append(['With V-to-V Charging', y, objective.value[()]])

  for t in range(12):
    constraints += [
      v[t] == 0
    ]

  problem = cp.Problem(cp.Minimize(objective), constraints)
  problem.solve(solver='ECOS')
  if objective.value is not None:
    results.append(['Without V-V Charging', y, objective.value[()]])



np.set_printoptions(suppress=True)
np.set_printoptions(precision=0)
results
```

```
Out[ ]: [['With V-to-V Charging', 100, 800.0000038077858],
         ['Without V-V Charging', 100, 800.0000000251299],
         ['With V-to-V Charging', 150, 700.0000001087831],
         ['Without V-V Charging', 150, 700.0000003218047],
         ['With V-to-V Charging', 200, 600.0000000331498],
         ['Without V-V Charging', 200, 600.0000000954607],
         ['With V-to-V Charging', 250, 500.0000007668473],
         ['Without V-V Charging', 250, 500.00000002615474],
         ['With V-to-V Charging', 300, 400.00000018794805],
         ['Without V-V Charging', 300, 399.99999998803577],
         ['With V-to-V Charging', 350, 300.00000000608105],
         ['Without V-V Charging', 350, 300.0000000030822],
         ['With V-to-V Charging', 400, 250.00000000518617],
         ['Without V-V Charging', 400, 300.000000000107],
         ['With V-to-V Charging', 450, 225.0000000037554],
         ['Without V-V Charging', 450, 300.0000000004379],
         ['With V-to-V Charging', 500, 225.00000002447143],
         ['Without V-V Charging', 500, 300.000000000391],
         ['With V-to-V Charging', 550, 225.00000005095708],
         ['Without V-V Charging', 550, 300.00000000045594],
         ['With V-to-V Charging', 600, 225.00000000818676],
         ['Without V-V Charging', 600, 300.000000000284],
         ['With V-to-V Charging', 650, 225.0000000019047],
         ['Without V-V Charging', 650, 300.00000000025807],
         ['With V-to-V Charging', 700, 225.00000000560817],
         ['Without V-V Charging', 700, 300.0000000002915],
         ['With V-to-V Charging', 750, 225.0000000127245],
         ['Without V-V Charging', 750, 300.00000000034646],
         ['With V-to-V Charging', 800, 225.0000000100056],
         ['Without V-V Charging', 800, 300.0000000004001],
         ['With V-to-V Charging', 850, 225.0000000032973],
         ['Without V-V Charging', 850, 300.00000000052523],
         ['With V-to-V Charging', 900, 225.00000008719323],
         ['Without V-V Charging', 900, 300.00000000059276],
         ['With V-to-V Charging', 950, 225.00000005957924],
         ['Without V-V Charging', 950, 300.0000000006417]]
```
